# Intimate Partner Violence Against Women and Mortality Among Children Younger Than 5 Years: A Systematic Review and Meta-Analysis

**DOI:** 10.1001/jamanetworkopen.2026.31794

**Published:** 2026-09-02

**Authors:** Bereket Kefale, Aditi Roy, Daniel Gashaneh Belay, Tesfaye Setegn Mengistu, Jennifer Dunne, Sylvester Dodzi Nyadanu, Amanuel T. Gebremedhin, Marshall Makate, Jacqueline Hendriks, Gavin Pereira, Gizachew A. Tessema

**Affiliations:** 1 Curtin School of Population Health, Curtin University, Perth, Australia; 2 Department of Reproductive and Family Health, School of Public Health, College of Medicine and Health Sciences, Wollo University, Dessie, Ethiopia; 3 Australian Research Council Centre of Excellence for the Elimination of Violence against Women, Monash University, Melbourne, Australia; 4 Department of Epidemiology and Biostatistics, Institute of Public Health, College of Medicine and Health Sciences, University of Gondar, Gondar, Ethiopia; 5 School of Public Health, Bahir Dar University, Bahir Dar, Ethiopia; 6 School of Public Health, The University of Queensland, Brisbane, Australia; 7 Charles Darwin University, Alice Springs Campus, Northern Territory, Australia; 8 enAble Institute, Curtin University, Perth, Australia; 9 School of Nursing and Midwifery, Edith Cowan University, Perth, Australia; 10 Nutrition and Health Innovation Research Institute, School of Medical and Health Sciences, Edith Cowan University, Perth, Australia; 11 WHO Collaborating Centre for Climate Change and Health Impact Assessment, Perth, Australia; 12 Faculty of Medicine, Universitas Negeri Malang, Malang, Indonesia.; 13 Institute for Health Research, University of Notre Dame, Fremantle, Australia; 14 School of Public Health, College of Health, Adelaide University, Adelaide, Australia

## Abstract

**Importance:**

Intimate partner violence (IPV) against women is a persistent global public health concern with lasting impacts on the health of affected women and their children. Although previous studies suggest a potential link between IPV and mortality among children younger than 5 years (under-5 mortality [U5M]), the evidence remains inconclusive.

**Objective:**

To examine whether different forms of IPV are associated with under-5, infant, and neonatal mortality.

**Data Sources:**

Seven databases (MEDLINE, EMBASE, Global Health, PsycINFO, Scopus, Web of Science, and CINAHL) were searched from database inception to April 22, 2026.

**Study Selection:**

Studies were included if they examined the association between forms of IPV (any IPV or subtypes such as physical, sexual, emotional violence, or partner control behavior) and under-5, infant, and neonatal mortality. Two reviewers independently conducted study selection.

**Data Extraction and Synthesis:**

The Preferred Reporting Items for Systematic Reviews and Meta-Analyses reporting guideline was followed. Risk of bias was assessed using the Risk of Bias in Non-randomized Studies of Exposures tool. Random-effects meta-analyses were conducted to estimate pooled odds ratios (ORs) and 95% CIs.

**Results:**

Twenty-eight studies comprising 684 065 participants were included in the systematic review, of which 25 were included in the meta-analyses. IPV was associated with higher odds of U5M (OR, 1.10; 95% CI, 1.05-1.16), infant mortality (OR, 1.32; 95% CI, 1.11-1.57), and neonatal mortality (OR, 1.36; 95% CI, 1.19-1.55). Physical IPV (OR, 1.37; 95% CI, 1.17-1.61), sexual IPV (OR, 1.70; 95% CI, 1.33-2.17), and emotional IPV (OR, 1.29; 95% CI, 1.07-1.56) were associated with higher odds of neonatal mortality. Physical IPV was also associated with higher odds of infant mortality (OR, 1.20; 95% CI, 1.01-1.44). No statistically significant associations were observed between IPV subtypes and U5M.

**Conclusions and Relevance:**

In this systematic review and meta-analysis, IPV against women was associated with higher odds of under-5, infant, and neonatal mortality. These findings suggest the importance of addressing IPV within child health strategies.

## Key Points

**Question:**

Is intimate partner violence against women associated with mortality among children younger than 5 years (under-5 mortality)?

**Findings:**

In this systematic review and meta-analysis of 28 studies including 684 065 participants, intimate partner violence against women was significantly associated with 10%, 32%, and 36% higher odds of under-5, infant, and neonatal mortality, respectively.

**Meaning:**

These findings suggest intimate partner violence against women is associated with an increased risk of under-5, infant, and neonatal mortality, highlighting the need to integrate intimate partner violence prevention into child survival strategies.

## Teaser

This systemic review and meta-analysis examines intimate partner violence against women and mortality among children younger than 5 years (under-5 mortality), infants, and neonates.

## Introduction

Intimate partner violence (IPV) against women is defined as behaviors within a relationship that result in physical, sexual, or psychological harm. It includes acts of physical aggression, sexual coercion, emotional abuse, and controlling behavior, perpetrated by current or former spouses or partners. IPV is a critical public health concern and a violation of fundamental human rights.^1,2^ Globally, more than one-quarter of ever-partnered women have experienced lifetime IPV, with prevalence varying across regions from 16% in Southern Europe to 57% in Oceania (excluding Australia and New Zealand).^3^ IPV has profound immediate and lasting consequences for women’s physical,^4^ mental,^5^ and sexual and reproductive health.^6^ IPV is also associated with increased risk of adverse maternal and perinatal outcomes, including physical injury to the fetus,^4^ maternal depression,^7^ inadequate weight gain,^8^ impaired maternal-infant attachment,^9^ low birth weight,^10^ preterm labor,^10^ miscarriage and unsafe abortion,^11^ stillbirth,^10^ and other obstetric complications.^12^

Emerging evidence suggests a potential link between IPV and child mortality.^13,14,15^ Proposed pathways include both direct effects (eg, violence during pregnancy) and indirect effects through adverse birth outcomes, compromised maternal health, and reduced access to care.^4,7,8,9,10,11,16,17,18^ These mechanisms may increase the risk of mortality among children younger than 5 years (under-5 mortality [U5M]); however, empirical evidence on this association remains limited and inconsistent.

Understanding the association between IPV and U5M is crucial for informing the design of effective interventions to address both immediate and long-term health consequences, thereby improving maternal and child health outcomes, and contributing to the attainment of sustainable development goals (SDGs) 3 (good health and well-being) and 5 (gender equality). While a systematic review in 2011 reported a statistically significant association between IPV and U5M,^15^ later studies have shown conflicting findings.^19,20,21^ Notably, the 2011 review neither evaluated the methodological quality of included studies nor investigated the specific forms of IPV or different child mortality outcomes. A recent umbrella review concluded that the current evidence on the association between IPV and U5M remains limited.^22^ Thus, this systematic review and meta-analysis was conducted to (1) quantify the association between IPV and under-5, infant, and neonatal mortality globally and (2) assess the association between IPV subtypes—physical, sexual, emotional IPV, and partner control behavior—and each child mortality outcome.

## Methods

### Search Strategy and Selection Criteria

We followed the Preferred Reporting Items for Systematic Reviews and Meta-Analyses (PRISMA) reporting guideline.^23^ The review protocol was registered in the International Prospective Register of Systematic Reviews (PROSPERO; CRD42023441477). We searched 7 databases: EMBASE, MEDLINE, Global Health, PsycINFO, Scopus, Web of Science, and CINAHL, as well as Google Scholar for gray literature. The reference lists of eligible studies were reviewed to identify additional articles. We included studies from database inception to December 24, 2023, and updated the search on November 9, 2025, and April 22, 2026, without geographic or language restrictions. Search terms included both subject headings (medical subject heading terms) and relevant keywords associated with IPV and child mortality outcomes (eTable 1 in Supplement 1). B.K. initially developed the search strategy, which was subsequently reviewed and approved by the research team.

Studies were eligible for inclusion following the Population, Exposure, Comparison, Outcome, and Study Design criteria. First, the population comprised women of reproductive age and their children younger than 5 years. Second, exposure criteria included forms of IPV (physical, emotional, sexual violence, or partner controlling behavior), as defined in the original studies, including exposure assessed during pregnancy or with no specified timing. Third, the comparison groups were women in the same study population who did not experience IPV. Fourth, the outcome of interest was all-cause mortality, including neonatal mortality (death within 28 days), infant mortality (death within 12 months), and U5M (death before age 5 years). Fifth, eligible study designs included quantitative observational studies, such as cohort, case-control, cross-sectional, and other epidemiological designs. We excluded studies that (1) were nonprimary or descriptive, including reviews, commentaries, editorials, case reports, and case series; and (2) did not assess the exposure-outcome association of interest.

All identified records were exported to EndNote 20 for deduplication and imported to Covidence for screening. Two reviewers (B.K. and T.S.M.) conducted screening, with disagreements resolved by a third reviewer (G.A.T.). For studies derived from the same cohort, we included the most recent publication, provided it had clearly defined exposure and outcome measurements.

### Data Extraction and Quality Appraisal

Two reviewers (B.K. and T.S.M.) independently extracted data from included studies using a predefined data extraction form. Extracted information included the first author, year of publication, country or region, study design, sample size, data sources, type of exposure, type of outcome, effect estimates with 95% CIs, method of exposure and outcome ascertainment, and adjustment variables. The risk of bias in the included studies was assessed by B.K. and D.G.B. using the risk of bias in nonrandomized studies of exposures tool.^24^ Any disagreements were resolved through discussion with a third reviewer (G.A.T.). Interrater agreement was substantial (Cohen κ = 0.68).^25^

### Statistical Analysis

Data were summarized using narrative synthesis, tables, and figures. Meta-analyses were conducted to estimate pooled odds ratios (ORs) for associations between different IPV subtypes and mortality outcomes. The analyses were conducted at multiple stages following each database search update (January 2023, November 2025, and April 2026). A random-effects meta-analysis with the restricted maximum likelihood estimator was used due to anticipated heterogeneity across study populations, settings, study designs, and IPV measurement methods. Studies reporting multiple IPV subtype-specific estimates without an overall estimate were first pooled using fixed-effects meta-analysis and then combined to generate overall pooled estimates for the association of any IPV and each mortality outcome. Statistical heterogeneity was assessed using the *I*^2^ statistic and Cochran *Q* test.^26^

Subgroup analyses were conducted to explore sources of heterogeneity using study-level characteristics, such as (1) sample size, (2) study design, (3) adjustment for confounding, and (4) risk of bias. To further investigate the sources of heterogeneity, we performed random-effects metaregression. Inverse variance–weighted random-effects cumulative meta-analysis (a sequential approach in which studies are added in chronological order and a new pooled estimate is recalculated at each step) was undertaken to assess changes in the pooled OR with the addition of new studies. The OR and 95% CI limits required (stability threshold) for a new study to move the cumulative OR to the null were reported.^27,28^ Leave-one-out sensitivity analyses were conducted to examine the influence of individual studies on the pooled estimates. Funnel plots and the Egger test were used to assess publication bias. Duval and Tweedie trim-and-fill analysis was applied to explore and adjust for publication bias.^29^ All analyses were conducted using Stata version 18 (StataCorp LLC).

## Results

**Figure 1.**
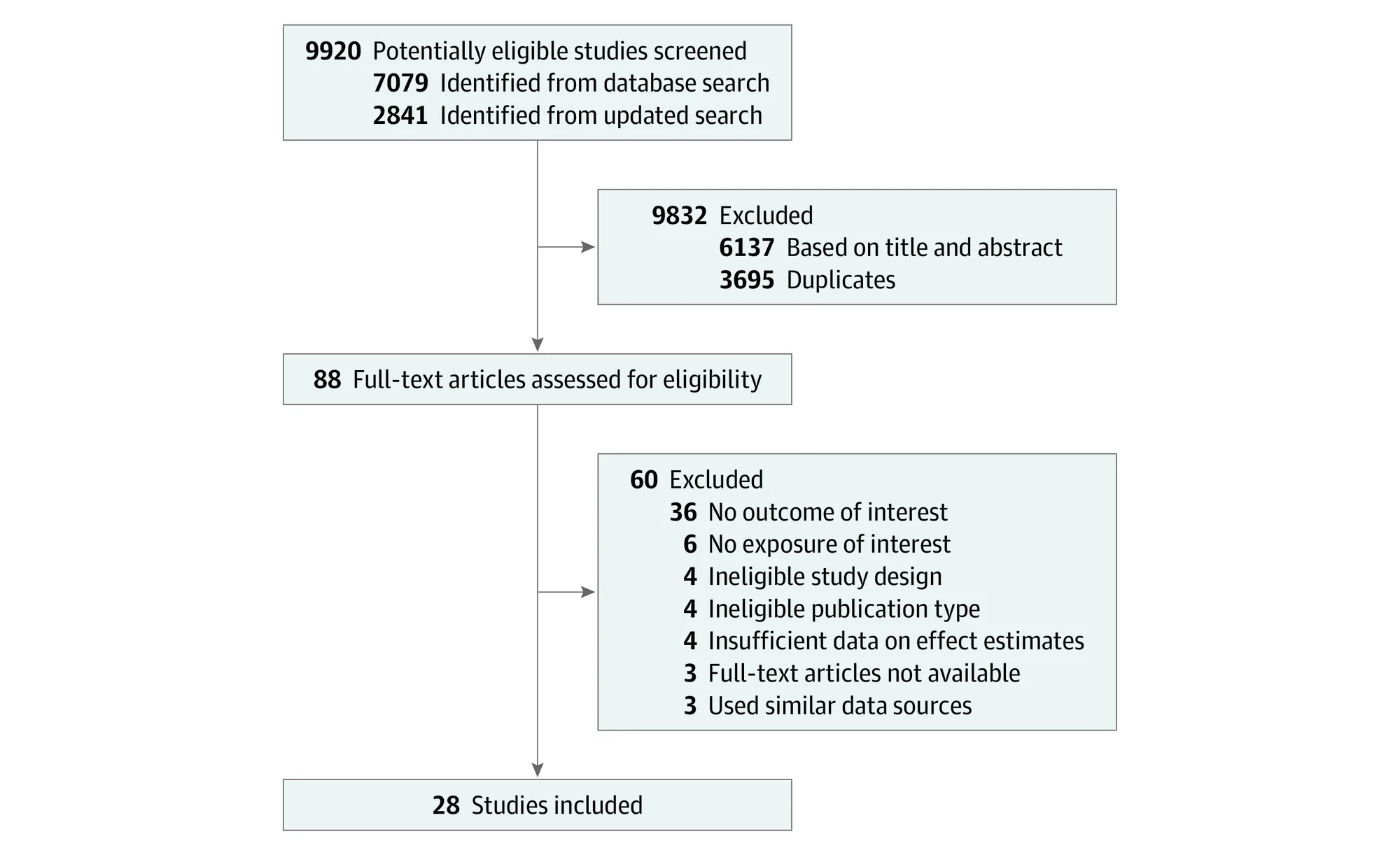
The PRISMA Flow Diagram PRISMA indicates Preferred Reporting Items for Systemic Reviews and Meta-Analyses reporting guidelines.

In total, 9920 records were identified from electronic databases and gray literature sources. We excluded 3695 duplicate articles and 6137 irrelevant articles based on title and abstract screening. Subsequently, 88 articles underwent full-text review, of which 28 fulfilled the eligibility criteria for inclusion in the final synthesis (Figure 1).

### Characteristics of Included Studies

Of the included 28 articles^13,14,19,20,21,30,31,32,33,34,35,36,37,38,39,40,41,42,43,44,45,46,47,48,49,50,51,52^ comprising a total of 684 065 participants, 26 studies (93%) originated from low- and middle-income countries,^13,14,19,20,30,31,32,33,34,35,36,37,38,39,40,41,42,43,44,45,46,47,48,49,50,51,52,53,54,55,56^ including 7 (25%) from India^30,39,40,43,44,45,52^ and 4 (14%) from Ethiopia.^36,37,46,50^ In terms of study design, 19 studies (68%) were cross-sectional,^13,14,19,30,33,34,35,38,39,40,41,42,45,48,49,50,51,52^ and 5 (18%) were cohort studies.^21,32,36,44,47^ The sample size of the included studies ranged from 160 participants^43^ to 227 121 participants.^14^ Sixteen studies (46%) examined 2 or more forms of IPV as separate exposures (Table 1).^19,20,30,32,33,35,36,37,38,39,40,41,42,43,44,45,46,47,48,49,50,51,52,53,54,55,56,57,3^

**Table 1.** Characteristics of Studies Included in the Systematic Review and Meta-Analysis (n = 28)

Source	Country	Study design	Data source	Study period	Sample size and study population	Exposure	Outcome
Ackerson et al,^30^ 2009	India	Cross-sectional	NFHS	2005-2006	39 096 Children aged <5 years	Any IPV, physical IPV, sexual IPV, emotional IPV	U5M, infant mortality
Ahmed et al,^45^ 2006	India	Cross-sectional	Women and the Male Reproductive Health Survey	1995-1996	2199 Pregnant women	Physical IPV in pregnancy	Neonatal mortality
Ashenafi et al,^46^ 2020	Ethiopia	Matched case-control	Community-based primary data	January to October 2018	515 (103 Mothers of deceased neonates, and 412 mothers of surviving neonates)	Physical IPV in pregnancy, sexual IPV in pregnancy, emotional IPV in pregnancy, partner control behavior in pregnancy	Neonatal mortality
Asling-Monemi et al,^31^ 2003	Nicaragua	Matched case-control	A demographic database consisted of population survey	January 1993 to June 1996	313 Children aged <5 years (110 deceased, and 203 alive)	Physical or sexual IPV	U5M, infant mortality
Asling-Monemi et al,^32^ 2008	Bangladesh	Cohort	Health and Demographic Surveillance System	July 1, 1982 to June 30, 2001	2691 Live-born children	Physical IPV, sexual IPV, emotional IPV, partner control behavior, IPV in pregnancy	U5M
Batool et al,^33^ 2018	Pakistan	Cross-sectional	DHS	2012-2013	3207 Married reproductive age women	Physical IPV, emotional IPV	U5M
Chidiebere et al,^34^ 2016	Nigeria, Egypt, Kenya, Democratic Republic of the Congo, Zimbabwe	Cross-sectional	DHS	2005-2008	3389 Women of reproductive age	Any IPV	U5M
Dadras et al,^35^ 2023	Afghanistan	Cross-sectional	DHS	2015	22 927 Children aged <5 years	Any IPV, physical IPV, sexual IPV, emotional IPV in the past 12 mo	U5M, infant mortality, neonatal mortality
Dendup et al,^20^ 2021	Bhutan	Cross-sectional	NHS	November 2012 to February 2013	2179 Births	Any IPV, IPV in pregnancy	Infant mortality
Deyessa et al,^36^ 2010	Ethiopia	Cohort	Community-based primary data	January to December 2002	561 Women with live births	Physical IPV in the past 12 mo, sexual IPV in the past 12 mo, emotional IPV in the past 12 mo	U5M
Garoma et al,^37^ 2012	Ethiopia	Case-control	Community-based primary data	May to June 2011	858 (286 Deceased [cases] and 572 alive [controls]) children aged <5 years	Any IPV, physical IPV, sexual IPV, emotional IPV, partner control behavior	U5M
Hossain et al,^38^ 2014	Bangladesh	Cross-sectional	DHS	March 24 to August 11, 2007	12 842 Children	Any IPV	U5M
Khatir et al,^51^ 2025	Afghanistan	Cross-sectional	DHS	2015	6494 Singleton births	Physical IPV	Neonatal mortality
Koenig et al,^44^ 2010	India	Prospective cohort	Primary follow-up data	1998-2003	3909 Births	Physical IPV in the past 12 mo	Neonatal mortality, Infant mortality
Liimatainen,^19^ 2021	Nigeria	Cross-sectional	DHS	August 14 to December 29, 2018	8389 Women of reproductive age	Any IPV, physical IPV, sexual IPV, emotional IPV	U5M
Lipsky et al,^47^ 2003	United States	Retrospective cohort	Birth and death records linked with police files	January 1995 to September 1999	3479 Births	Any IPV in pregnancy, physical IPV in pregnancy	Neonatal mortality
Lockington et al,^21^ 2023	Australia	Retrospective cohort	Women’s electronic health records	January 2017 to December 2021	45 177 Births	Any IPV	Neonatal mortality
Memiah et al,^13^ 2020	East Africa	Cross-sectional	DHS	2014-2016	11 512 Women of reproductive age	Any IPV	U5M, infant mortality, and neonatal mortality
Paul et al,^39^ 2020	India	Cross-sectional	NFHS	2015-2016	34 317 Mother-children’s pairs	Physical IPV, sexual IPV, emotional IPV	U5M, infant mortality
Pool et al,^48^ 2014	Ghana	Cross-sectional	DHS	2008	1745 Women	Physical IPV in pregnancy	Neonatal mortality
Roy et al,^52^ 2026	India	Cross-sectional	NFHS	2015-2016 and 2019-2021	58 685 Mother-child pairs	Any IPV	U5M, infant mortality
Shamu et al,^49^ 2018	Zimbabwe	Cross-sectional	Institutional-based primary data	2011	2042 Women of reproductive age	Physical IPV, sexual IPV, emotional IPV	Neonatal mortality
Silverman et al,^40^ 2011	India	Cross-sectional	NFHS	November 2005 to August 2006	158 439 Women of reproductive age	Any IPV	U5M, infant mortality
Taft et al,^41^ 2015	Timor-Leste	Cross-sectional	DHS	January 1 to May 31, 2004	1959 Ever-married women of reproductive age	Any IPV, physical IPV	U5M
Tiruye et al,^50^ 2021	Ethiopia	Cross-sectional	DHS	January 18 to June 27 2016	2863 Currently married women of reproductive age	Any IPV, physical IPV, sexual IPV, emotional IPV, partner control behavior	Neonatal mortality
Titilayo et al,^42^ 2017	Nigeria	Cross-sectional	DHS	2013	26 997 Ever-married women	Physical IPV, sexual IPV, emotional IPV	U5M
Varghese et al,^43^ 2013	India	Matched case-control	A central register a comprehensive community health program	January 2000 to December 2006	160 (80 Deceased [cases], and 80 alive [controls]) children aged <5 years	Any IPV, IPV in pregnancy	U5M
Yaya et al,^14^ 2020	Sub-Saharan Africa	Cross-sectional	DHS	2010-2018	227 121 Women of reproductive age	Any IPV	U5M

Abbreviations: DHS, Demographic and Health Survey; IPV, intimate partner violence; NFHS, National Family Health survey; NHS, National Health Survey; U5M, under-5 mortality.

### Risk of Bias

Four studies that did not adjust for confounding were rated as having a very high risk of bias.^32,36,42,47^ Six studies were deemed to have a high risk of bias.^31,33,37,44,49,51^ The remaining 18 studies had some concerns. All studies had at least some concerns regarding participant selection and exposure measurement (eFigures 1 and 2 in Supplement 1).

### Intimate Partner Violence and U5M

**Figure 2.**
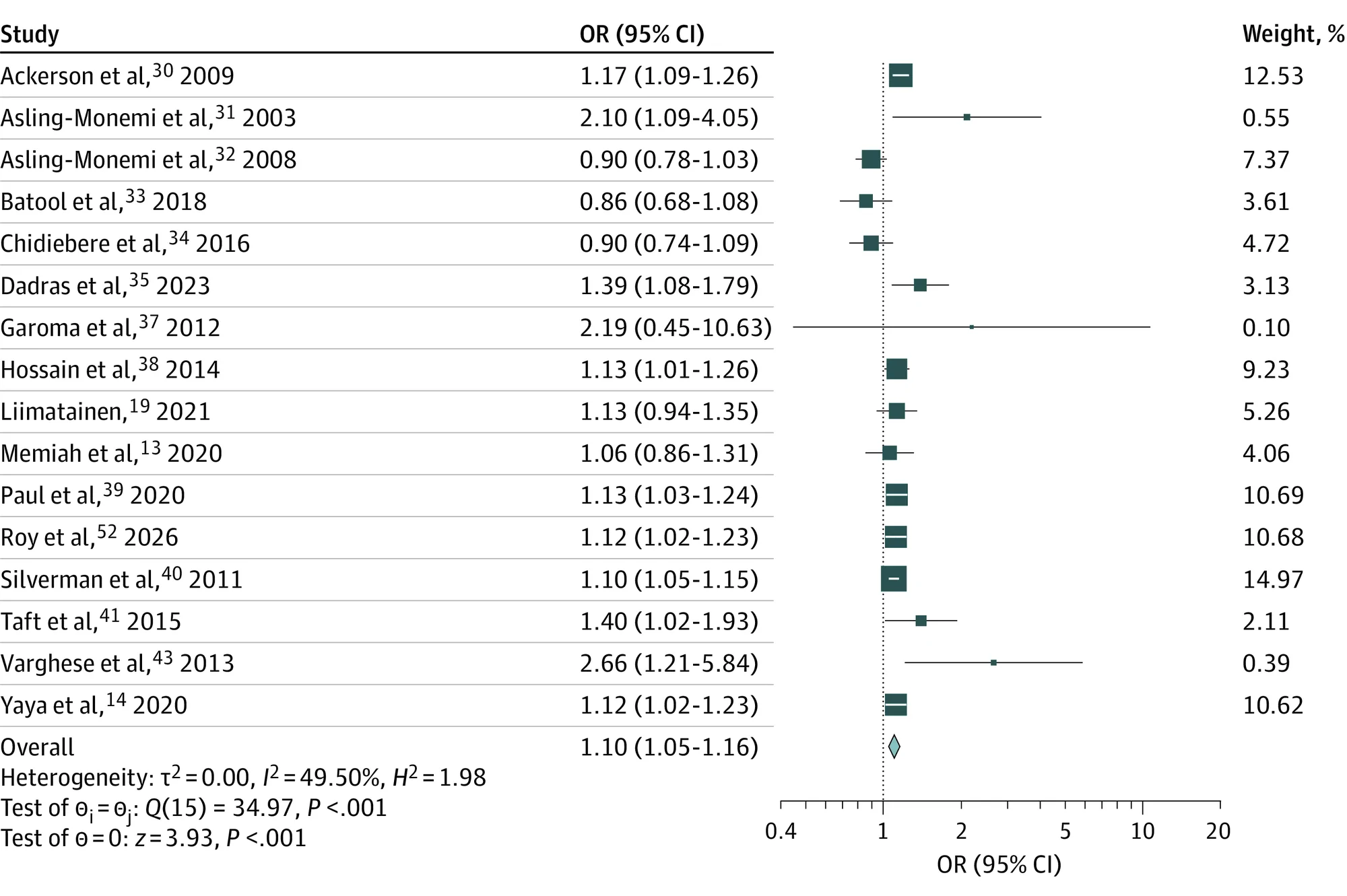
Forest Plot Showing the Association Between Any Intimate Partner Violence and Under-5 Mortality OR indicates odds ratio.

Eighteen studies^13,14,19,30,31,32,33,34,35,36,37,38,39,40,41,42,43,52^ examined the association between IPV and U5M. Two studies^36,42^ that did not report adjusted estimates were excluded from the meta-analysis. A meta-analysis of 16 studies (n = 585 905) showed 10% higher odds of U5M associated with maternal IPV (OR, 1.10; 95% CI, 1.05-1.16; I^2^ = 50%) (Figure 2).

We found no statistically significant associations between specific types of IPV and U5M, including physical IPV (8 studies; OR, 1.12; 95% CI, 0.99-1.26; I^2^ = 61%), sexual IPV (6 studies; OR, 1.21; 95% CI, 0.95-1.56; I^2^ = 83%), emotional IPV (7 studies; OR, 1.05; 95% CI, 0.95-1.15; I^2^ = 0%), and partner control behaviors (2 studies; OR, 1.75; 95% CI, 0.47-6.47; I^2^ = 69%). No statistically significant association was observed between IPV during pregnancy and U5M (2 studies; OR, 1.99; 95% CI, 0.52-7.61; I^2^ = 88%) (Table 2; eFigures 3 and 4 in Supplement 1).

**Table 2.** Associations of Intimate Partner Violence Subtypes With Under-5, Infant, and Mortality Among Children Younger Than 5 Years

Outcome	Studies, No.	Sample size	OR (95% CI)	*I*^2^, %	*P* value (*Q* test)
Under-5 mortality					
Any IPV	16	585 905	1.10 (1.05-1.16)	50	<.001
Physical IPV	8	113 444	1.12 (0.99-1.26)	61	.09
Sexual IPV	6	108 278	1.21 (0.95-1.56)	83	<.001
Emotional IPV	7	111 485	1.05 (0.95- 1.15)	0	.37
Partner control behavior	2	3549	1.75 (0.47-6.47)	69	.07
IPV during pregnancy	2	2851	1.99 (0.52-7.61)	88	<.001
Infant mortality					
Any IPV	9	331 377	1.32 (1.11-1.57)	89	<.001
Physical IPV	4	100 249	1.20 (1.01-1.44)	53	.09
Sexual IPV	3	96 340	1.26 (0.95-1.69)	59	.08
Emotional IPV	3	96 340	1.11 (0.91-1.37)	32	.29
Neonatal mortality					
Any IPV	11	155 053	1.36 (1.19-1.55)	37	.03
Physical IPV	8	39 679	1.37 (1.17-1.61)	0	.42
Sexual IPV	4	28 347	1.70 (1.33-2.17)	0	.51
Emotional IPV	4	28 347	1.29 (1.07-1.56)	0	.24
Partner control behavior	2	3378	1.58 (0.68-3.68)	51	.15
IPV during pregnancy	4	7938	1.93 (1.43-2.60)	0	.41

Abbreviations: *I*^2^, heterogeneity statistic; IPV, Intimate partner violence; OR, odds ratio; Q test, Cochran’s *Q* test for heterogeneity.

### Intimate Partner Violence and Infant Mortality

**Figure 3.**
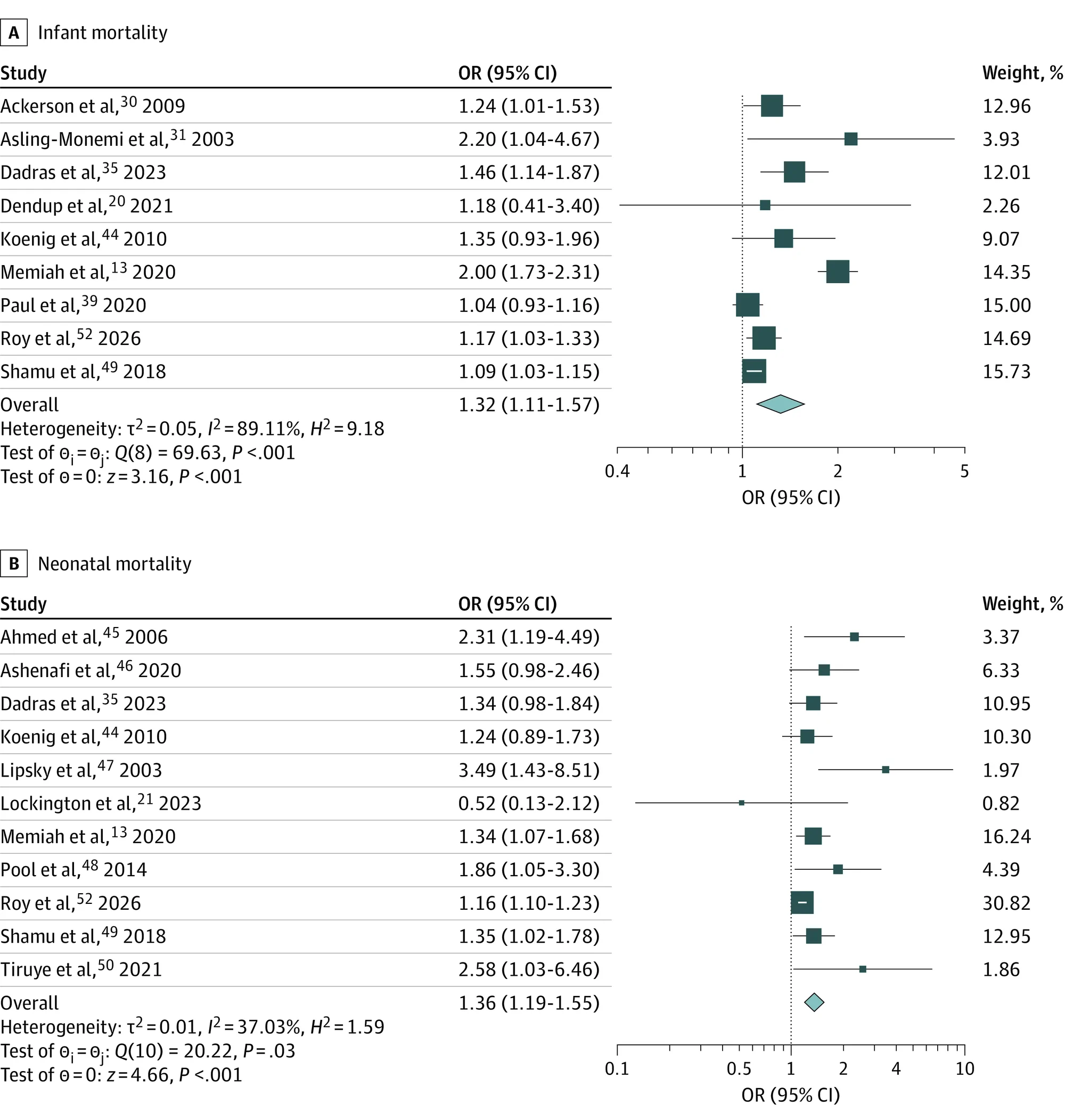
Forest Plots Showing the Association Between Any Intimate Partner Violence and Infant and Neonatal Mortality OR indicates odds ratio.

Nine studies^13,20,30,31,35,39,40,44,52^ (n = 331 377) examined the association between IPV and infant mortality. The pooled estimate indicated 32% higher odds of infant mortality among women experiencing IPV (OR, 1.32; 95% CI, 1.11-1.57; I^2 =^  89%) (Figure 3). Exposure to physical IPV was associated with a 20% increase in the odds of infant mortality (OR, 1.20; 95% CI, 1.01-1.44; I^2^ = 53%). The associations for sexual IPV and emotional IPV were not statistically significant (Table 2; eFigure 5 in Supplement 1).

### Intimate Partner Violence and Neonatal Mortality

Twelve studies^13,21,35,44,45,46,47,48,49,50,51,52^ (n = 161 547) examined the association between IPV and neonatal mortality. One study was excluded from the meta-analysis due to inconsistently reported effect estimates.^51^ In a pooled analysis of 11 studies, neonates born to mothers exposed to IPV had 36% higher odds of neonatal mortality (OR, 1.36; 95% CI, 1.19-1.55; I^2^ = 37%) (Figure 3). Women exposed to physical IPV had 37% higher odds of neonatal mortality (OR, 1.37; 95% CI, 1.17-1.61; I^2^ = 0%). Sexual IPV was associated with 70% higher odds of neonatal mortality (OR, 1.70; 95% CI, 1.33-2.17; I^2^ = 0%). Emotional IPV was associated with 29% higher odds of neonatal mortality (OR, 1.29; 95% CI, 1.07-1.56; I^2^ = 0%). IPV during pregnancy was associated with 93% higher odds of neonatal mortality (OR, 1.93; 95% CI, 1.43-2.60). Nevertheless, the association between partner control behavior and neonatal mortality was not statistically significant (Table 2; eFigures 6 and 7 in Supplement 1).

### Publication Bias

The funnel plot for studies examining any IPV and U5M demonstrated asymmetry, suggesting potential publication bias, supported by Egger test (*P* = 0.03). A trim-and-fill analysis imputed 4 potentially missing studies, yielding a similar effect size (OR, 1.09; 95% CI, 1.03-1.15). For studies examining the association between IPV and neonatal mortality, the funnel plot suggested asymmetry, and the Egger test provided statistically significant evidence of small-study effects (*P* = 0.001). A trim-and-fill analysis, which imputed 3 studies, yielded an effect estimate consistent with the primary analysis (OR, 1.28; 95% CI, 1.14-1.43) (eFigure 8 in Supplement 1).

### Subgroup and Sensitivity Analyses

Heterogeneity was observed among studies pooled for the association between IPV and U5M (I^2^ = 50%). Subgroup analyses among studies with large sample sizes (OR, 1.10; 95% CI, 1.04-1.15) and cross-sectional studies (OR, 1.12; 95% CI, 1.08-1.15) yielded results consistent with the overall estimate. However, studies at high risk of bias found no statistically significant association (OR, 1.12; 95% CI, 0.72-1.73) (eTable 2 in Supplement 1). Metaregression indicated that sample size and risk of bias were significant sources of heterogeneity (eTable 3 in Supplement 1). Estimates derived from studies published after 2016, together with stability thresholds, demonstrated minimal variation in the pooled OR. The final stability threshold for U5M was an OR of 0.91 (upper 95% CI; limit = 1.03): a new observational study would need to report a protective assocation of this magnitude, with precision similar to that of the 16 pooled studies, to move the pooled estimate to the null. The findings were robust to sensitivity analyses, which involved removing one study at a time (eTable 4, eFigures 9 and 10 in Supplement 1).

## Discussion

This systematic review and meta-analysis found higher odds of under-5, infant, and neonatal mortality among children of women who experienced IPV. Physical IPV was associated with higher odds of infant and neonatal mortality, while sexual IPV and emotional IPV were associated with higher odds of neonatal mortality. However, the associations between specific IPV subtypes and U5M were not statistically significant.

Our findings on the association between any IPV and U5M align with a previous systematic review, which also reported a higher risk of U5M among children of women exposed to IPV.^15^ The observed association was highest for neonatal mortality, followed by infant and U5M, which may reflect age-specific pathways linking IPV to child survival. Neonatal mortality is likely associated with prenatal and perinatal factors. For example, IPV during pregnancy may adversely affect fetal development through maternal stress, physical injury, inadequate antenatal care, and poor maternal nutrition, leading to adverse birth outcomes such as preterm birth and low birth weight, which are key determinants of neonatal mortality.^10^ For infant and U5M, the associations may be shaped by postnatal caregiving environments, including maternal capacity to provide adequate feeding, hygiene, and timely health care seeking for the child, while still reflecting perinatal and early neonatal determinants.^53,54^ Infants and young children in IPV-affected households may therefore be particularly vulnerable to preventable injuries and infections when caregiving is compromised.^55^

The subtype-specific findings further support the association between IPV and child mortality. Physical IPV was associated with higher odds of infant and neonatal mortality, while sexual IPV was associated with higher odds of neonatal mortality. The increased odds of neonatal mortality associated with physical IPV may reflect severe maternal harm or death during the puerperal period, leaving newborns without primary caregivers at critical stages of life.^56^ Maternal stress and trauma associated with physical IPV may also compromise fetal development and neonatal survival.^57^ The higher odds of infant mortality among children of women exposed to physical IPV may indicate that its influence extends beyond the neonatal period, affecting caregiving practices, child nutrition, access to health care, and overall well-being.^58,59^ Furthermore, children in IPV-affected households may face increased risk of direct abuse or neglect, heightening their vulnerability to mortality.^60^ Persistent IPV may also be associated with maternal filicide,^61^ as it can impair a mother’s ability to cope with stress, increasing the risk of direct harm to the child or neglect.^62^

The increased odds of neonatal mortality among women exposed to sexual IPV may be explained by several interrelated factors. Sexual IPV during pregnancy has been associated with heightened maternal stress, contributing to adverse pregnancy outcomes such as preterm birth and low birth weight, both of which increase the risk of early childhood mortality.^63^ Women who experience sexual IPV may also encounter barriers to accessing maternal and child health services, elevating the risk of adverse birth outcomes.^64^ Furthermore, the psychological and social consequences of sexual IPV may impair a mother’s ability to provide consistent care for her child, further increasing mortality risk.^65^ Women’s exposure to emotional IPV was also associated with neonatal mortality. Emotional IPV may contribute to maternal malnutrition, depression, and compromised immune function, which adversely affect neonatal health outcomes. Additionally, women experiencing IPV may be less likely to seek timely and adequate prenatal care, increasing the risk of adverse birth outcomes and early childhood mortality.^10,58,66^

The associations between specific types of IPV and U5M were not statistically significant. The significant association observed between any IPV and U5M may reflect the cumulative impact of women being exposed to multiple IPV forms, whereas the nonsignificant findings for each specific type likely reflect a limited number of studies and reduced statistical power.

We observed moderate heterogeneity among studies examining the association between IPV and U5M. This heterogeneity may reflect contextual differences in cultural norms that influence IPV disclosure, variability in IPV measurement tools, differences in confounding adjustment and recall periods, and broader socioeconomic and health system differences. Although subgroup analyses and metaregression were limited by the number of available studies, the overall pooled estimate was consistent with findings from studies with larger sample sizes.

Metaregression analysis showed that study size was associated with differences in effect estimates across studies, with smaller studies (less than 1000 participants) reporting higher effect estimates. This pattern may reflect small-study factors, including publication bias, residual confounding, selective reporting, and greater sampling variability, which can lead to more unstable and sometimes more extreme estimates. Smaller studies also tend to yield less precise effect estimates, which may limit the ability to detect systematic differences across studies, thereby masking true between-study heterogeneity.^26,67,68^ In addition, studies with a high risk of bias contributed to heterogeneity, potentially reflecting unmeasured confounding and exposure misclassification.

### Strengths and Limitations

This study has strengths. We conducted a comprehensive search across seven major databases without geographic or language restrictions and applied rigorous methodologies to synthesize evidence on the association between IPV and child mortality. We explored sources of heterogeneity through multiple subgroup analyses and metaregression.

However, some inherent limitations of this study should be acknowledged. Most included studies were conducted in low- and middle-income countries, limiting the generalizability of findings to high-income countries. Approximately two-thirds of the studies used cross-sectional designs, restricting causal inference. Many relied on retrospective reporting of lifetime IPV exposure, which may introduce recall bias, while social desirability bias may also have led to underreporting of IPV due to social stigma or fear of disclosure. Although adjusted estimates were used, some studies accounted for a limited number of covariates, raising the possibility of residual confounding. Finally, the limited number of studies for each child mortality outcome and IPV subtype, together with evidence of publication bias and heterogeneity, reduces certainty in these associations.

## Conclusions

In this systematic review and meta-analysis, we found that IPV against women was associated with higher odds of under-5, infant, and neonatal mortality. These findings highlight the importance of addressing IPV within child health strategies, including strengthening legal protections, expanding survivor support systems, and integrating IPV detection and response into maternal and child health services. Future large-scale, prospective studies, particularly from high-income settings, are needed to strengthen the evidence base and to clarify the pathways through which IPV influences child survival outcomes. In addition, future research may use more granular, nonoverlapping age groups, particularly separating the postneonatal period and ages 1 to 4 years, to examine age-specific associations.

## Notes

Supplement 1. eTable 1. Search Term and Strategy eTable 2. Subgroup Analyses for the Association of Intimate Partner Violence and Under-5 Mortality eTable 3. Univariate and Multivariate Meta-Regression Analyses for the Association Between Intimate Partner Violence and Under-5 Mortality eTable 4. Cumulative Odds Ratios for the Association Between Intimate Partner Violence and Under-5 Mortality with Stability Thresholds eTable 5. List of Confounders Included in the Analysis of the Association Between Intimate Partner Violence and Under-5 Mortality eTable 6. Effect Estimates for the Association Between Intimate Partner Violence and Child Mortality Outcomes in the Included Studies eTable 7. List of Excluded Studies After Full-text Review eFigure 1. Risk of Bias of Included Studies Assessed Using the Risk of Bias in Nonrandomized Studies of Exposures Tool eFigure 2. Risk of Bias of Each Included Studies Assessed Using the Risk of Bias in Nonrandomized Studies of Exposures Tool eFigure 3. Forest Plots of the Associations Between Physical (A), Sexual (B), and Emotional (C) Intimate Partner Violence with Under-5 Mortality eFigure 4. Forest Plots of the Associations Between Partner Control Behavior (A) and Intimate Partner Violence During Pregnancy (B) and Under-5 Mortality eFigure 5. Forest Plots of the Associations Between Physical (A), Sexual (B), and Emotional (C) Intimate Partner Violence with Infant Mortality eFigure 6. Forest Plots of the Associations Between Physical (A), Sexual (B), and Emotional (C) Intimate Partner Violence with Neonatal Mortality eFigure 7. Forest Plots of the Associations Between Partner Control Behavior (A) and Intimate Partner Violence During Pregnancy (B) and Neonatal Mortality eFigure 8. Funnel Plots in Assessing Publication Bias in Studies of Intimate Partner Violence and Under-5 Mortality (A) and Neonatal Mortality (B) eFigure 9. Cumulative Meta-Analysis of the Association Between Any Intimate Partner Violence and Under-5 Mortality eFigure 10. Leave-One-Out Sensitivity Analysis of the Association Between Any Intimate Partner Violence And Under-5 (A), Infant (B), and Neonatal Mortality (C)

Supplement 2. Data Sharing Statement
