# Supplementary material for: Intimate Partner Violence Against Women and Mortality Among Children Younger Than 5 Years: A Systematic Review and Meta-Analysis
Source: JAMA Netw Open. 2026 Sep 2;9(9):e2631794. doi: 10.1001/jamanetworkopen.2026.31794 (PMC13539397; doi:10.1001/jamanetworkopen.2026.31794)
Supplement: Supplementary file 1 [file jamanetwopen-e2631794-s001.pdf]

Supplemental Online Content

Kefale B, Roy A, Belay DG, et al. Intimate partner violence against women and childhood mortality. *JAMA Netw Open.* 2026;9(9):e2631794.
doi:10.1001/jamanetworkopen.2026.31794

eTable 1. Search Term and Strategy

eTable 2. Subgroup Analyses for the Association of Intimate Partner Violence and Under-5 Mortality

eTable 3. A Univariate and Multivariate Meta-Regression Analyses for the Association Between Intimate Partner Violence and Under-Five Mortality

eTable 4. Cumulative Odds Ratios for the Association Between Intimate Partner Violence and Under-Five Mortality with Stability Thershold

eTable 5. List of Confounders Included in the Analysis of the Association Between Intimate Partner Violence and Under-Five Mortality

eTable 6. Effect Estimates for the Association Between Intimate Partner Violence and Child Mortality Outcomes in the Included Studies

eTable 7. List of Excluded Studies After Full-text Review

eFigure 1. Risk of Bias of Included Studies Assessed Using the Risk of Bias in Non-randomized Studies of Exposures Tool

eFigure 2. Risk of Bias of Each Included Studies Assessed Using the Risk of Bias in Non-randomized Studies of Exposures Tool

eFigure 3. Forest Plots of the Associations Between Physical (A), Sexual (B), and Emotional (C) Intimate Partner Violence with Under-Five Mortality

eFigure 4. Forest Plots of the Associations Between Partner Control Behavior (A) and Intimate Partner Violence During Pregnancy (B) and Under-Five Mortality

eFigure 5. eFigure 5. Forest Plots of the Associations Between Physical (A), Sexual (B), and Emotional (C) Intimate Partner Violence with Infant Mortality

eFigure 6. Forest Plots of the Associations Between Physical (A), Sexual (B), and Emotional (C) Intimate Partner Violence with Neonatal Mortality

eFigure 7. Forest Plots of the Associations Between Partner Control Behavior (A) and Intimate Partner Violence During Pregnancy (B) and Neonatal Mortality

eFigure 8. Funnel Plots in Assessing Publication Bias in Studies of Intimate Partner Violence and Under-five Mortality (A) and Neonatal Mortality (B)

eFigure 9. Cumulative Meta-Analysis of the Association Between Any Intimate Partner Violence and Under-5 Mortality

© 2026 Kefale B et al. *JAMA Network Open*.

eFigure 10. Leave-One-Out Sensitivity Analysis of the Association Between Any Intimate Partner Violence And Under-5 (A), Infant (B), and Neonatal Mortality (C)

This supplemental material has been provided by the authors to give readers additional information about their work.

© 2026 Kefale B et al. *JAMA Network Open*.

eTable 1. The Search Strategy and Search Term

Databases	s.no	Headings, key terms with proximity operators
**Embase (Ovid)**	#1	exp violence/ OR exp abuse/ OR exp coercion/OR exp harassment/ OR exp assault/
	#2	(violence OR abuse OR coercion OR harassment OR assault OR rape OR "partner* control*" or "battered women"). ti,ab.
	#3	#1 or #2
	#4	exp perinatal mortality/ or exp perinatal death/ or exp newborn mortality/ or exp newborn death/ or exp infant mortality/ or exp child death/ or exp childhood mortality/
	#5	((perinatal or new-born* or neonat* or infant or child* or under-five or under-5 or "under five" or "under 5" or baby or toddler* or paediatric*) adj2 (mortalit* or death*)).ti,ab.
	#6	#4 or #5
	#7	#3 and #6
	#8	limit 7 to (human and (article or article in press or "preprint)
**Medline (Ovid)**	#1	exp Violence/ or exp Coercion/ or exp Sexual Harassment/ or exp Battered Women/
	#2	(violence OR abuse OR coercion OR harassment OR assault OR rape OR "partner* control*" OR "battered women"). ti,ab.
	#3	#1 or #2
	#4	exp Perinatal Death/ or exp Infant Mortality/ or exp Infant Death/ or exp Child Mortality/
	#5	((perinatal or new-born* or neonat* or infant or child* or under-five or under-5 or "under five" or "under 5" or baby or toddler* or paediatric*) adj2 (mortalit* or death*)).ti,ab.
	#6	#4 or #5
	#7	#3 and #6
	#8	Limit 7 to (humans and (journal article or preprint))
**Global Health (Ovid)**	#1	exp abuse/ OR exp sexual harassment/ OR exp sexual assault/ OR exp rape/ OR exp sexual assault/ OR exp intimate partner violence/
	#2	(violence OR abuse OR coercion OR harassment OR assault OR rape OR "partner* control*" or "battered women"). ti,ab.
	#3	#1 or #2
	#4	exp perinatal mortality/ or exp neonatal mortality/ or exp infant mortality/
	#5	((perinatal or new-born* or neonat* or infant or child* or under-five or under-5 or "under five" or "under 5" or baby or toddler* or paediatric*) adj2 (mortalit* or death*)).ti,ab.
	#6	#4 or #5
	#7	#3 and #6
	#8	Limit 7 to (Journal article or preprint or thesis))
**PsycINFO (Ovid)**	#1	exp Violence/ OR exp Emotional Abuse/ OR exp Physical Abuse/ OR exp Verbal Abuse/ OR exp Sexual Abuse/ OR exp Coercion/ OR exp Harassment/
	#2	(violence OR abuse OR coercion OR harassment OR assault OR rape OR "partner* control*" or "battered women"). ti,ab.
	#3	#1 or #2
	#4	exp child death/
	#5	((perinatal or new-born* or neonat* or infant or child* or under-five or under-5 or "under five" or "under 5" or baby or toddler* or paediatric*) adj2 (mortalit* or death*)).ti,ab.
	#6	#4 or #5
	#7	#3 and #6
	#8	Limit 7 to (humans and (dissertation or journal article or reprint))
**Scopus**	#1	TITLE-ABS-KEY (violence OR abuse OR coercion OR harassment OR assault OR rape OR "partner* control*" OR "battered women")
	#2	TITLE-ABS-KEY ("perinatal death*" OR "new-born* death*" OR "neonat* death*" OR "infant death*" OR "child* death*" OR "under-five death*" OR "under-5 death*" OR "under five death*" OR "under 5 death*" OR "baby death*" OR "toddler* death*" OR "paediatric* death*" OR "perinatal mortalit*" OR "new-born*

© 2026 Kefale B et al. *JAMA Network Open*.

		mortalit*" OR "neonat* mortalit*" OR "infant mortalit*" OR "child* mortalit*" OR "under-five mortalit*" OR "under-5 mortalit*" OR "under five mortalit*" OR "under 5 mortalit*" OR "baby death*" OR "toddler* mortalit*" OR "paediatric* mortalit*")
	#3	#1 and #2
	#4	LIMIT-TO (DOCTYPE , "ar") AND (LIMIT-TO (EXACTKEYWORD , "human"))
**Web of science**	#1	TI=((violence OR abuse OR coercion OR harassment OR assault OR rape OR "partner* control*" OR "battered women")) OR AB=((violence OR abuse OR coercion OR harassment OR assault OR rape OR "partner* control*" or "battered women"))
	#2	TI=(("perinatal death*" or "new-born* death*" or "neonat* death*" or "infant death*" or "child* death*" or "under-five death*"or "under-5 death*"or "under five death*" or "under 5 death*" or "baby death*" or "toddler* death*" or "paediatric* death*" or "perinatal mortalit*" or "new-born* mortalit*" or "neonat* mortalit*" or "infant mortalit*" or "child* mortalit*" or "under-five mortalit*" or "under-5 mortalit*"or "under five mortalit*" or "under 5 mortalit*" or "baby death*" or "toddler* mortalit*" or "paediatric* mortalit*")) OR AB=(("perinatal death*" or "new-born* death*" or "neonat* death*" or "infant death*" or "child* death*" or "under-five death*"or "under-5 death*"or "under five death*" or "under 5 death*" or "baby death*" or "toddler* death*" or "paediatric* death*" or "perinatal mortalit*" or "new-born* mortalit*" or "neonat* mortalit*" or "infant mortalit*" or "child* mortalit*" or "under-five mortalit*" or "under-5 mortalit*"or "under five mortalit*" or "under 5 mortalit*" or "baby death*" or "toddler* mortalit*" or "paediatric* mortalit*"))
	#3	#1 and #2
	#4	#3 limited Article (Document Types)
**CINAHL (EBSCO)**	#1	(MH "Violence+") OR (MH "Coercion") OR (MH "Sexual Harassment") OR (MH "Sexual Abuse+") OR (MH "Gender-Based Violence") OR (MH "Emotional Abuse") OR (MH "Battered Women")
	#2	TI (violence OR abuse OR coercion OR harassment OR assault OR rape OR "partner* control*" OR "battered women") OR AB (violence OR abuse OR coercion OR harassment OR assault OR rape OR "partner* control*" or "battered women")
	#3	#1 and #2
	#4	(MH "Perinatal Death") OR (MH "Infant Mortality") OR (MH "Child Mortality")
	#5	TI ((perinatal or new-born* or neonat* or infant or child* or under-five or under-5 or "under five" or "under 5" or baby or toddler* or paediatric*) N2 (mortalit* or death*)) OR AB ((perinatal or new-born* or neonat* or infant or child* or under-five or under-5 or "under five" or "under 5" or baby or toddler* or paediatric*) N2 (mortalit* or death*))
	#6	#4 or #5
	#7	#3 and #6
**Google Scholar**		(Violence|abuse|assault|rape|coercion|harassment|"partner control") ("neonatal mortality"|"infant mortality"|"child mortality"|"under-five mortality")

© 2026 Kefale B et al. *JAMA Network Open*.

eTable 2. Sub-Group Analyses for the Association of Intimate Partner Violence and Under-Five Mortality

Variables	No. of studies	Sample size	OR (95% CI)	I^2 (%)	p-value (Q test)
**Study sample size**					
<1000	3	1331	2.30 (1.42-3.72)[a]	0	0.90
≥1000	13	584,574	1.10 (1.04-1.15)[a]	53	<0.001
**Study design**					
Cross-sectional	12	581,883	1.12 (1.08-1.15)[a]	0	0.11
Case-control or cohort	4	4,022	1.61 (0.87-2.99)	73	<0.001
**Adjusted for maternal age**					
Yes	12	355,620	1.12 (1.08-1.15)[a]	0	0.09
No	4	230,285	1.37 (0.86-2.18)	96	<0.001
**Adjusted for wealth status**					
Yes	14	584,734	1.10 (1.05-1.15)[a]	52	<0.001
No	2	1,171	2.11 (1.15-3.88)[a]	0	0.96
**Adjusted for place of residence**					
Yes	7	496,752	1.11 (1.07-1.14)[a]	0	0. 32
**No**	9	89,153	1.14 (0.98-1.33)	76	<0.001
**Adjusted for maternal employment status**					
Yes	7	137,120	1.09 (1.01-1.16)[a]	47	<0.001
No	9	448,785	1.18 (1.04-1.35)[a]	83	<0.001
**Risk of bias**					
Some concerns	11	578,836	1.12 (1.09-1.15)[a]	0	0.12
High or very high risk	4	7,069	1.12 (0.72-1.73)	88	0.05
Total	15	585,905	1.10 (1.05-1.16)[a]	50	<0.001

[a]p-value less than 0.001; CI, confidence interval; I^2, heterogeneity statistic; OR, odds ratio; Q-test, Cochran's Q test for heterogeneity.

© 2026 Kefale B et al. *JAMA Network Open*.

eTable 3. A Univariate and Multivariate Meta-Regression Analyses for the Association Between Intimate Partner Violence and Under-Five Mortality

Variables	Crude OR (95% CI)	p-value	Adjusted OR (95% CI)	p-value
**Sample size**				
≥1000	Ref		Ref	
<1000	2.10 (1.29-3.43)	0.003	2.32 (1.40-3.85)	0.001
**Study design**				
Cross-sectional	Ref		Ref	
Case-control or cohort	0.87 (0.76-0.99)	0.037	1.04 (0.80-1.36)	0.74
**Risk of bias**				
Some concerns	Ref		Ref	
High risk	0.82 (0.73-0.92)	0.001	0.77 (0.61-0.97)	0.023

CI, confidence interval; OR, odds ratio; Ref, reference category.

© 2026 Kefale B et al. *JAMA Network Open*.

eTable 4. Cumulative Odds Ratios for the e Association Between Intimate Partner Violence and Under-Five Mortality with Stability Thershold

Author (Year)	Year	OR (95% CI)	Cumulative OR (95% CI)	Stability Threshold (Pereira)	I^2 (%)
Asling-Monemi et al,[1] 2003	2003	2.10 (1.10, 4.10)	2.10 (1.09, 4.05)	0.48 (CI: 1.27)	0
Asling-Monemi et al,[2] 2008	2008	0.90 (0.79, 1.04)	1.29 (0.57, 2.93)	0.78 (CI: 2.48)	83.6
Ackerson et al,[3] 2009	2009	1.17 (1.09, 1.26)	1.17 (0.81, 1.69)	0.85 (CI: 1.44)	93.2
Silverman et al,[4] 2011	2011	1.10 (1.05, 1.15)	1.10 (0.93, 1.29)	0.91 (CI: 1.14)	89.4
Garoma et al,[5] 2012	2012	2.19 (0.45, 10.60)	1.10 (0.94, 1.29)	0.91 (CI: 1.13)	86.7
Varghese et al,[6] 2013	2013	2.66 (1.21, 5.83)	1.26 (0.95, 1.66)	0.80 (CI: 1.19)	95.5
Hossain et al,[7] 2014	2014	1.13 (1.01, 1.26)	1.13 (1.00, 1.27)	0.89 (CI: 1.05)	80.0
Taft et al,[8] 2015	2015	1.40 (1.02, 1.93)	1.16 (1.02, 1.33)	0.86 (CI: 1.05)	83.4
Chidiebere et al,[9] 2016	2016	0.90 (0.74, 1.09)	1.12 (0.99, 1.27)	0.89 (CI: 1.06)	82.9
Batool et al,[10] 2018	2018	0.86 (0.68, 1.08)	1.09 (0.97, 1.23)	0.91 (CI: 1.08)	82.9
Memiah et al,[11] 2020	2020	1.06 (1.01, 1.55)	1.08 (0.98, 1.20)	0.92 (CI: 1.06)	75.8
Paul et al,[12] 2020	2020	1.13 (1.03, 1.24)	1.09 (1.00, 1.18)	0.92 (CI: 1.03)	70.9
Yaya et al,[13] 2020	2020	1.12 (1.02, 1.23)	1.09 (1.02, 1.16)	0.92 (CI: 1.03)	63.7
Liimatainen,[14] 2021	2021	1.13 (0.95, 1.36)	1.09 (1.03, 1.16)	0.92 (CI: 1.03)	56.4
Dadras et al,[15] 2023	2023	1.39 (1.08, 1.79)	1.10 (1.04, 1.18)	0.91 (CI: 1.03)	61.7
Roy et al,[16] 2026	2026	1.12 (0.98, 1.18)	1.10 (1.05, 1.16)	0.91 (CI: 1.03)	49.6

CI: 95% confidence interval limit; I^2, heterogeneity statistic; OR, odds ratio;

© 2026 Kefale B et al. *JAMA Network Open*.

eTable 5. List of Confounders Included in the Analysis of the Association Between Intimate Partner Violence and Under-Five Mortality

Study	List of confounders adjusted
Ackerson et al,[3] 2009	Maternal age, mother's age at birth, maternal autonomy, mother's height, mother's occupation, mother's education, father's education, wealth, caste, religion, household toilet, household drinking water source, household location, state, child sex, birth order.
Ahmed et al,[17] 2006	Women's age, parity, women's education, husband's education, socioeconomic status, caste, child sex, pregnancy status, prenatal care, tetanus toxoid immunization, place of delivery, and postpartum care
Ashenafi et al,[18] 2020	Age, maternal education status, paternal education status, no of under-five children, maternal occupation status, paternal occupation status, empowerment status, social support, alcohol intake, ANC utilization, PNC utilization, multiple pregnancy, place of birth, birth size, gravidity, parity, early breastfeeding initiation
Asling-Monemi et al,[1] 2003	Maternal educational status, residence, parity
Asling-Monemi et al,[2] 2008	Asset score, religion
Batool et al,[10] 2018	Maternal age, region, maternal educational status, wealth status, women work status, husband educational status, antenatal care utilization
Chidiebere et al,[9] 2016	Educational status, age, wealth index, occupation, literacy, residence, marital status, media access, decision autonomy, number of Children
Dadras et al,[15] 2023	Maternal age, education, place of living, wealth index, and ethnicity, age of marriage, ANC visits, child age, and child sex
Dendup et al,[19] 2021	Maternal age, age at first marriage, age at first pregnancy, marital status, child sex, total birth, sex of household head, birth order, birth interval, maternal education, husband education, household head education, household size, maternal working status, household working status, wealth index, safe drinking water, sanitation facility availability, use of solid fuel, residence, region, contraceptive use, place of delivery, skilled delivery, antenatal care, postnatal care, colostrum feeding
Deyessa et al,[20] 2009	Not stated
Garoma et al,[5] 2012	Age of mother, education, parity, birthweight, alcohol
Hossain et al,[7] 2014	Age of mother, residence, education, region, occupation, wealth index, number of children, access to media, decision autonomy
Khatir et al,[21] 2025	
Koenig et al,[22] 2010	Maternal age, maternal education, paternal education, household assets, caste, prenatal care, child sex, parity, state
Liimatainen,[14] 2021	Residence, religion, maternal age, educational status, work status, wealth
Lipsky et al,[23] 2003	Marital status
Lockington et al,[24] 2023	Socioeconomic status, ethnicity, refugee status
Memiah et al,[11] 2020	Maternal age, residence, wealth index, maternal age at first birth, religion, educational status, working status, husband educational level, mass media use, desire for pregnancy, maternal BMI, sexual autonomy, maternal giving sexual Favors, delivery by caesarean section, delivery assistance, place of delivery, antenatal care, contraceptive use, husband age, husband working status, child sex, size of child at birth, birth weight, birth order, birth interval
Paul et al,[12] 2020	Child sex, birth order, age of mother, maternal age at marriage, maternal education, father's education, maternal BMI, maternal anaemia, place of residence, caste, religion, wealth quintile

© 2026 Kefale B et al. *JAMA Network Open*.

Pool et al,[25] 2014	Maternal age, total children ever born, level of education, wealth index, current marital status, place of residence
Roy et al,[16] 2026	
Shamu et al,[26] 2018	Maternal age, education, marital status, and HIV
Silverman et al,[4] 2011	Maternal age, education, parity, residence, and household wealth index
Taft et al,[8] 2015	Maternal age, age at marriage, educational level, region, wealth index, place of residence, family type
Tiruye et al,[27] 2021	Respondent age at birth, respondent's educational status, access to media, household wealth index, region of residence, residence, respondent's substance abuse, number of living children, pregnancy status
Titilayo et al[28] 2017	Maternal age, maternal education, residence, region, wealth index, age of marriage
Varghese et al,[6] 2013	Socioeconomic status, maternal and paternal literacy, at least one boy child in family, paternal alcohol abuse, paternal tobaco abuse, immunisation completed for age
Yaya et al,[13] 2020	Survey year, educational status, wealth index, maternal decision-making power, parity, polygamy, residence, gender index, Human Development Index

© 2026 Kefale B et al. *JAMA Network Open*.

eTable 6. Effect Estimates for the Association Between Intimate Partner Violence and Child Mortality Outcomes in the Included Studies

Study(year)	Mortality outcome	IPV type	Unadjusted effect size measure (95% CI)	Adjusted effect size measure (95% CI)
Ackerson et al,[3] 2009	Under-five mortality	Any IPV	NA	RR 1.17 (1.09, 1.26)
		Physical IPV	NA	RR 1.21 (1.10, 1.32)
		Sexual IPV	NA	RR 0.85 (0.64, 1.11)
		Emotional IPV	NA	RR 1.06 (0.87, 1.30)
	Infant mortality	Any IPV	NA	RR 1.24 (1.01, 1.53)
		Physical IPV	NA	RR 1.05 (0.79, 1.41)
		Sexual IPV	NA	RR 1.46 (0.86, 2.46)
		Emotional IPV	NA	RR 1.02 (0.57, 1.81)
Ahmed et al,[17] 2006	Neonatal mortality	Physical IPV in pregnancy	HR 1.92 (1.00, 3.68)	HR 2.31 (1.19, 4.50)
	Post neonatal mortality	Physical IPV in pregnancy	HR 1.03 (0.46, 2.30)	HR 1.00 (0.44.2.27)
	Child mortality	Physical IPV in pregnancy	HR 1.38 (0.22, 8.77)	HR 0.94 (0.12, 7.15
Ashenafi et al,[18] 2020	Neonatal mortality	Physical IPV in pregnancy (moderate)	OR 1.17 (0.41. 3.29)	OR 0.96 (0.28, 3.16)
		Physical IPV in pregnancy (severe)	OR 1.30 (0.23, 7.24)	OR 0.99 (0.12, 7.95)
		Sexual IPV in pregnancy	OR 2.98 (1.05, 8.43)	OR 3.20 (1.09, 9.33)
		Emotional IPV in pregnancy	OR 1.18 (0.60, 2.32)	OR 0.87 (0.38, 1.96)
		Partner controlling behavior in pregnancy	OR 2.36 (1.06, 5.24)	OR 2.42 (1.06, 5.51)
Asling-Monemi et al,[1] 2003	Under-five mortality	Physical IPV	OR 4.3 (1.9–9.5)	NA
		Sexual IPV	OR 3.2 (1.6–6.3)	NA
		Physical or sexual IPV	NA	OR 2.10 (1.10, 4.10)
		Physical IPV in the past 12 months	OR 1.9 (0.7, 4.8)	NA
	Infant mortality	Physical IPV	OR 2.1 (1.1, 4.0)	NA
		Sexual IPV	OR 2.2 (1.2, 3.9)	NA
		Physical or sexual IPV	NA	OR 2.20 (1.00, 4.50)
		Physical IPV in the past 12 months	OR 1.6 (0.5, 4.7)	NA
Asling-Monemi et al,[2] 2008	U5M	Physical IPV (daughter educated)	NA	HR 1.00 (0.54, 2.01)
		Physical IPV (daughter uneducated)	NA	HR 1.00 (0.64, 1.48)
		Physical IPV (boy educated)	NA	HR 0.80 (0.40, 1.44)
		Physical IPV (boy uneducated)	NA	HR 0.70 (0.42, 1.13)
		Sexual IPV (daughter educated)	NA	HR 0.90 (0.49, 1.79)
		Sexual IPV (daughter uneducated)	NA	HR 0.90 (0.60, 1.38)
		Sexual IPV (boy educated)	NA	HR 1.10 (0.59, 2.04)
		Sexual IPV (boy uneducated)	NA	HR 0.80 (0.49, 1.28)
		Emotional IPV (daughter educated)	NA	HR 1.10 (0.56, 2.30)

© 2026 Kefale B et al. *JAMA Network Open*.

		Emotional IPV (daughter uneducated)	NA	HR 0.80 (0.54, 1.33)
		Emotional IPV (boy educated)	NA	HR 0.70 (0.43, 1.76)
		Emotional IPV (boy uneducated)	NA	HR 0.80 (0.48, 1.38)
		Partner control behavior (daughter educated)	NA	HR 2.50 (1.30, 4.90)
		Partner control behavior (daughter uneducated)	NA	HR 0.90 (0.55, 1.55)
		Partner control behavior (boy educated)	NA	HR 1.00 (0.45, 2.00)
		Partner control behavior (boy uneducated)	NA	HR 1.00 (0.58, 1.81)
		Physical IPV in pregnancy (daughter educated)	NA	HR 2.10 (0.87, 5.11)
		Physical IPV in pregnancy (daughter uneducated)	NA	HR 0.90 (0.44, 1.66)
		Physical IPV in pregnancy (boy educated)	NA	HR 1.20 (0.41, 3.34)
		Physical IPV in pregnancy (boy uneducated)	NA	HR 0.70 (0.32, 1.51)
Batool et al,[10] 2018	U5M	Any IPV	OR 1.28 (1.05, 1.57)	NA
		Physical IPV	OR 1.40 (1.13, 1.73)	OR 0.75 (0.51,1.09)
		Emotional IPV	OR 1.36 (1.12, 1.67)	OR 0.93 (0.69, 1.24)
Chidiebere et al,[9] 2016	U5M	Any IPV (Nigeria)	NA	OR 0.71 (0.53, 0.94)
		Any IPV (Egypt)	NA	OR 0.36 (0.42, 2.2)
		Any IPV (Kenya)	NA	OR 2.58 (0.90, 7.33)
		Any IPV (Democratic Republic of Congo)	NA	OR 1.04 (0.86, 1.90)
		Any IPV Zimbabwe)	NA	OR 1.29 (0.87, 1.91)
Dadras et al,[15] 2023	U5M	Any IPV	OR 1.47 (1.11, 1.94)	OR 1.39 (1.08, 1.79)
		Physical IPV	OR 1.52 (1.18, 1.95)	OR 1.40 (1.11, 1.77)
		Sexual IPV	OR 1.57 (1.09, 2.27)	OR 1.45 (1.03, 2.05)
		Emotional IPV	OR 1.32 (0.98, 1.77)	OR 1.26 (0.98, 1.64)
	Infant mortality	Any IPV	OR 1.54 (1.16, 2.04)	OR 1.46 (1.14, 1.87)
		Physical IPV	OR 1.59 (1.24, 2.04)	OR 1.47 (1.18, 1.85)
		Sexual IPV	OR 1.64 (1.12, 2.42)	OR 1.53 (1.07, 2.21)
		Emotional IPV	OR 1.36 (1.01, 1.84)	OR 1.31 (1.00, 1.70)
	Neonatal mortality	Any IPV	OR 1.40 (1.00, 1.95)	OR 1.34 (0.98, 1.85)
		Physical IPV	OR 1.45 (1.09, 1.93)	OR 1.36 (1.02, 1.83)
		Sexual IPV	OR 1.48 (0.93, 2.22)	OR 1.41 (0.94, 2.11)
		Emotional IPV	OR 1.23 (0.86, 1.75)	OR 1.18 (0.86, 1.62)
Dendup et al,[19] 2021	Infant mortality	Any IPV	HR 1.58 (0.56. 4.44)	HR 1.18 (0.41, 3.40)
		IPV in pregnancy	HR 4.09 (1.18, 14.17)	HR 3.76 (1.13, 12.56)
Deyessa et al,[20] 2009	U5M	Physical IPV in the past 12 months	RR 1.4 (0.7, 2.3)	NA
		Sexual IPV in the past 12 months	RR 1.2 (0.7, 1.9)	NA
		Emotional IPV in the past 12 months	RR 1.4 (0.8, 2.4)	NA
Garoma et al,[5] 2012	U5M	Any IPV	OR 2.41 (1.65, 3.52)	OR 2.19 (0.45, 10.60)

© 2026 Kefale B et al. *JAMA Network Open*.

		Physical IPV	OR 2.37 (1.63, 3.43)	OR 1.62 (0.15, 2.46)
		Sexual IPV	OR 1.94 (1.37, 2.74)	OR 1.63 (0.17, 2.33)
		Emotional IPV	OR 2.36 (1.62, 3.44)	OR 1.97 (0.44, 8.87)
		Partner control behavior	OR 1.80 (1.27, 2.55)	OR 4.27 (0.97, 18.89)
Hossain et al,[7] 2014	U5M	Any IPV	HR 1.21 (1.09, 1.35)	HR 1.13 (1.01, 1.26)
Khatir et al,[21] 2025	Neonatal mortality	Less severe physical IPV	HR 1.88	HR 1.99
		Severe physical IPV	HR 2.19	HR 2.39
Koenig et al,[22] 2010	Infant mortality	Physical IPV in the past 12 months (one violent episode)	NA	HR 0.59 (0.25, 1.31)
		Physical IPV in the past 12 months (more than one violent episode)	NA	HR 1.68 (1.10, 2.57)
	Neonatal mortality	Physical IPV in the past 12 months (one violent episode)	NA	HR 0.78 (0.45, 1.34)
		Physical IPV in the past 12 months (more than one violent episode)	NA	HR 1.62 (1.11, 2.53)
Liimatainen,[14] 2021	U5M	Any IPV	OR 1.15 (0.97, 1,37)	OR 1.13 (0.95, 1.36)
		Physical IPV	OR 0.99 (0.80, 1,22)	OR 1.09 (0.87. 1.35)
		Sexual IPV	OR 1.53 (1.16, 2,02)	OR 1.63 (1.22, 2.17)
		Emotional IPV	OR 1.16 (0.98, 1,40)	OR 1.11 (0.93, 1.34)
Lipsky et al,[23] 2003	Neonatal mortality	Any IPV in pregnancy	NA	OR 3.49 (1.43, 8.50)
		Physical IPV in pregnancy	NA	OR 2.85 (1.02, 7.98)
Lockington et al,[24] 2023	Neonatal mortality	Any IPV	OR 0.50 (0.12, 1.99)	OR 0.52 (0.13, 2.16)
Memiah et al,[11] 2020	U5M	Any IPV	OR 1.98 (1.73, 2.11)	OR 1.06 (1.01, 1.55)
	Infant mortality	Any IPV	OR 1.89 (1.64, 1.93)	OR 2.00 (1.66, 2.22)
	Neonatal mortality	Any IPV	OR 1.11 (0.74, 1.67)	OR 1.34 (1.4, 2.22)
Paul et al,[12] 2020	U5M	Physical IPV	OR 1.26 (1.10, 1.44)	OR 1.07 (0.93, 1.23)
		Sexual IPV	OR 1.17 (0.90, 1.51)	OR 1.32 (1.11, 1.56)
		Emotional IPV	OR 1.26 (1.06, 1.49)	OR 1.04 (0.86, 1.25)
	Infant mortality	Physical IPV	OR 1.25 (1.09, 1.44)	OR 1.07 (0.92, 1.24)
		Sexual IPV	OR 1.20 (0.91, 1.57)	OR 0.98 (0.73, 1.30)
		Emotional IPV	OR 1.23 (1.02, 1.47)	OR 1.01 (0.83, 1.23)
Pool et al,[25] 2014	Neonatal mortality	Physical IPV in pregnancy	NA	1.86 (1.05, 3.30)
Roy et al,[16] 2026	U5M	Any IPV	1.26 (1.12, 1.42)	1.12 (0.98, 1.18)
	Infant mortality	Any IPV	1.36 (1.09, 1.40)	1.17 (1.03, 1.33)
	Neonatal mortality	Any IPV	1.29 (1.09, 1.45)	1.16 (1.10, 1.23)
Shamu et al,[26] 2018		Physical IPV	NA	OR 1.17 (1.16, 2.20)

© 2026 Kefale B et al. *JAMA Network Open*.

	Neonatal mortality	Sexual IPV	NA	OR 1.80 (1.29, 2.49)
		Emotional IPV	NA	OR 1.34 (1.03, 1.72)
Silverman et al,[4] 2011	U5M	Any IPV	HR 1.39 (1.33, 1.46)	HR 1.10 (1.05, 1.15)
	Infant mortality	Any IPV	HR 1.34 (1.27, 1.41)	HR 1.09 (1.03, 1.15)
Taft et al,[8] 2015	U5M	Any IPV*	NA	OR 1.40 (1.02, 1.93)
		Physical IPV	NA	OR 1.26 (1.02, 1.56)
Tiruye et al,[27] 2021	Neonatal mortality	Any IPV	NA	OR 2.58 (1.03, 6.45)
		Physical IPV	NA	OR 1.35 (0.49, 3.68)
		Sexual IPV	NA	OR 1.85 (0.61, 5.57)
		Emotional IPV	NA	OR 2.92 (1.15, 7.38)
		Partner control behavior	NA	OR 1.02 (0.44, 2.39)
Titilayo et al,[28] 2017	U5M	Physical IPV	NA	OR 0.83
		Sexual IPV	NA	OR 0.88
		Emotional IPV	NA	OR 0.99
Varghese et al,[6] 2013	U5M	Any IPV	2.63 (1.39, 4.99)	2.66 (1.21, 5.83)
		IPV in pregnancy	5.69 (2.03, 15.93)	4.15 (1.80, 9.55)
Yaya et al,[13] 2020	U5M	Mild IPV	NA	1.04 (0.90, 1.19)
		Severe IPV	NA	1.19 (1.06, 1.34)

CI, confidence interval; HR, hazard ratio; IPV, intimate partner violence; NA, not available; OR, odds ratio; RR, relative risk; U5M, under-five mortality.

© 2026 Kefale B et al. *JAMA Network Open*.

eTable 7. List of Excluded Studies After Full-text Review

Author	Title	Reasons
Afkhamzadeh et al,[29] 2021	Adverse perinatal outcomes of intimate partner violence during pregnancy	No outcome of interest
Alhusen et al,[30] 2015	Intimate Partner Violence During Pregnancy: Maternal and Neonatal Outcomes	Ineligible study design (review article)
Alio et al,[31] 2009	Spousal violence and potentially single and recurrent spontaneous foetal loss in an African setting: cross-sectional study	No outcome of interest
Ara et al,[32] 2023	Sociodemographic determinants of child mortality based on mothers' attitudes toward partner violence: Evidence from Bangladesh	No exposure of interest (The violence perpetuated was not only by intimate partners)
Baird et al,[33] 2025	Domestic and family violence and associated maternal and perinatal outcomes: A population-based retrospective cohort study	No outcome of interest
Ball,[34] 2010	Intimate partner violence against mothers associated with child death in India	Full-text not available
Bari et al,[35] 2019	Physical Violence during Pregnancy and Infant Mortality: Evidence from Pakistan Demographic and Health Survey 2012-13	Ineligible publication type (conference abstract)
Berenson et al,[36] 1994	Perinatal morbidity associated with violence experienced by pregnant women	No outcome of interest
Bhatti et al,[37] 2017	Risk factors of neonatal mortality in Faisalabad, Pakistan	No exposure of interest
Butchart et al,[38] 2003	Violence against women and the risk of infant and child mortality	Ineligible publication type (commentary)
Chen et al,[39] 2017	Birth outcomes in relation to intimate partner violence	No outcome of interest
Coker et al,[40] 2004	Partner violence during pregnancy and risk of adverse pregnancy outcomes	No outcome of interest
Covington et al,[41] 2001	Preterm delivery and the severity of violence during pregnancy	No outcome of interest
Deutsch et al,[42] 2025	Social Factors Associated with Sudden Unexpected Infant Death	No outcome of interest
Devineni et al,[43] 2018	Effects of intimate partner violence on pregnancy outcome	No outcome of interest
El Kady et al,[44] 2005	Maternal and neonatal outcomes of assaults during pregnancy	No exposure of interest (The violence was not only perpetuated by intimate partners)
Eno et al,[45] 2014	Domestic violence and obstetric outcome among pregnant women in Ilorin, north central Nigeria	No exposure of interest (Physical violence was not reported in the model)
Fay et al,[46] 2020	Birth Outcomes Among Women Affected by Reproductive Coercion	No outcome of interest
Ghotbizadeh et al,[47] 2023	The Association Between Intimate Partner Violence During Pregnancy and Maternal and Neonatal Outcomes: A Cross-Sectional Study	No outcome of interest
Global Alliance for Nursing and Midwifery,[48] 2018	Gender-Based Violence and Child Mortality: they are related.	Ineligible publication type (blog article/post)
Gulliver et al,[49] 2014	Immediate and long-term outcomes of assault in pregnancy	No outcome of interest
Heaton et al,[50] 2008	Domestic violence, couple interaction and children's health in Latin America	Insufficient data on effect estimates
Ibrahimi et al,[51] 2021	Association of Domestic Physical Violence with feto-Infant Outcomes in Afghanistan	Used similar data source with Dadras et al., 2023
Janssen et al,[52] 2003	Intimate partner violence and adverse pregnancy outcomes: a population-based study	No outcome of interest

Jejeebhoy,[53] 1998	Associations between wife-beating and foetal and infant death: impressions from a survey in rural India	No outcome of interest
Jofre-Bonet et al,[54] 2024	Intimate partner violence and children's health outcomes	No outcome of interest
Karamagi et al,[55] 2007	Intimate partner violence and infant morbidity: Evidence of an association from a population-based study in eastern Uganda in 2003	No outcome of interest
Koirala,[56] 2022	Intimate partner violence and adverse pregnancy outcomes	No outcome of interest
McClintock et al,[57] 2023	Intimate partner violence and child loss: an evaluation of 7 sub-Saharan African countries	No outcome of interest
McDougal et al,[58] 2013	Adverse Pregnancy Outcomes and Sexual Violence Among Female Sex Workers Who Inject Drugs on the United States–Mexico Border	No outcome of interest
Melaku et al,[59] 2023	The Association Between Domestic Violence and Adverse Pregnancy Outcomes in Pregnant Women Seeking Antenatal Care at Public Hospitals in Gedeo Zone: A Prospective Cohort Study	No outcome of interest
Menon,[60] 2014	Unfinished Lives: The effect of domestic violence on neonatal and infant mortality	Insufficient data on effect estimates
Musa et al,[61] 2021	The association between intimate partner violence and low birth weight and preterm delivery in eastern Ethiopia: Findings from a facility-based study	No outcome of interest
Nassaka,[62] 2023	Influence of social economic status components on gender-based violence and infant mortality in Uganda	No outcome of interest
Nwachuku,[63] 2001	Infant and child mortality and related religion-cultural violence against women	Full-text not available
Okenwa et al,[64] 2011	Contraception, reproductive health, and pregnancy outcomes among women exposed to intimate partner violence in Nigeria	Used similar data source with Chidiebere et al., 2016
Okunola et al,[65] 2021	Adverse birth outcomes among women exposed to intimate partner violence in pregnancy in Ikere-Ekiti, South-west Nigeria: A prospective cohort study	No outcome of interest
Osuorah et al[66] 2012	Effect of maternal exposure to intimate partner violence on under-five mortality in Nigeria	Used similar data source with Ackerson et al., 2009
Pandey et al,[67] 2013	Adjusted Effects of Domestic Violence, Tobacco use, and Indoor Air Pollution from Use of Solid Fuel on Child Mortality	No exposure of interest (The exposure was the attitude towards partner violence)
Pastor-Moreno,[68] 2020	Intimate partner violence during pregnancy and a risk of foetal and neonatal death: a meta-analysis with socioeconomic context indicators	Ineligible study design (review article)
Rao et al,[69] 2017	Correlations between intimate partner violence and spontaneous abortion, stillbirth, and neonatal death in rural Malawi	No outcome of interest
Rawlings et al,[70] 2020	Domestic violence and child mortality in the developing world	Insufficient data on effect estimates
Rico et al,[71] 2011	Associations between maternal experiences of intimate partner violence and child nutrition and mortality: findings from Demographic and Health Surveys in Egypt, Honduras, Kenya, Malawi and Rwanda. Journal of Epidemiology & Community Health. 2011;65(4):360-7	No outcome of interest
Sabri et al,[72] 2023	Impact of domestic violence on maternal and child health and well-being in rural India	No outcome of interest
Sarkar,[72] 2008	The impact of intimate partner violence on women's reproductive health and pregnancy outcome	Ineligible study design (review article)
Sarkar,[73] 2013	The cause and consequence of domestic violence on pregnant women in India	Ineligible study design (review article)
Schoeps et al,[74] 2007	Risk factors for early neonatal mortality	No outcome of interest

© 2026 Kefale B et al. *JAMA Network Open*.

Schuiling,[74] 2016	Disrespect and Abuse of Women During Childbirth: A Significant Factor in the Efforts to Reduce Maternal and Perinatal Morbidity and Mortality	Ineligible publication type (editorial)
Shelton et al,[75] 2009	Spousal violence and spontaneous foetal loss	No outcome of interest
Silverman et al,[76] 2007	Intimate partner violence and unwanted pregnancy, miscarriage, induced abortion, and stillbirth among a national sample of Bangladeshi women	No outcome of interest
Silverman et al,[77] 2009	Maternal experiences of intimate partner violence and child morbidity in Bangladesh: Evidence from a national Bangladeshi sample	No outcome of interest
Sunitha,[78] 2008	Domestic Violence and The Risk of Infant and Child Mortality-A Case Control Study	Full-text not available
Tamirat et al,[79] 2021	Determinants of adverse birth outcome in Sub-Saharan Africa: analysis of recent demographic and health surveys	No outcome of interest
Valadbeigi et al,[80] 2017	The relationship between physical violence during pregnancy and stillbirth and neonatal mortality	No exposure of interest
Wassie et al[81],2023	The impact of intimate partner violence on adverse birth outcomes in public health facilities. A prospective cohort study	No outcome of interest
Weitzman et al,[82] 2020	The Marital Implications of Bereavement: Child Death and Intimate Partner Violence in West and Central Africa	No outcome of interest
Yadav et al,[83] 2017	A study on relationship between domestic violence and childhood mortality in India: Using Weibull frailty modelling approach	Insufficient data on effect estimates
Yost et al,[84] 2005	A prospective observational study of domestic violence during pregnancy	No outcome of interest
Yu et al,[85] 2018	Association of intimate partner violence during pregnancy, prenatal depression, and adverse birth outcomes in Wuhan, China	No outcome of interest
Zakar et al,[86] 2016	The association of intimate partner violence with unintended pregnancy and pregnancy loss in Pakistan	No outcome of interest

© 2026 Kefale B et al. *JAMA Network Open*.

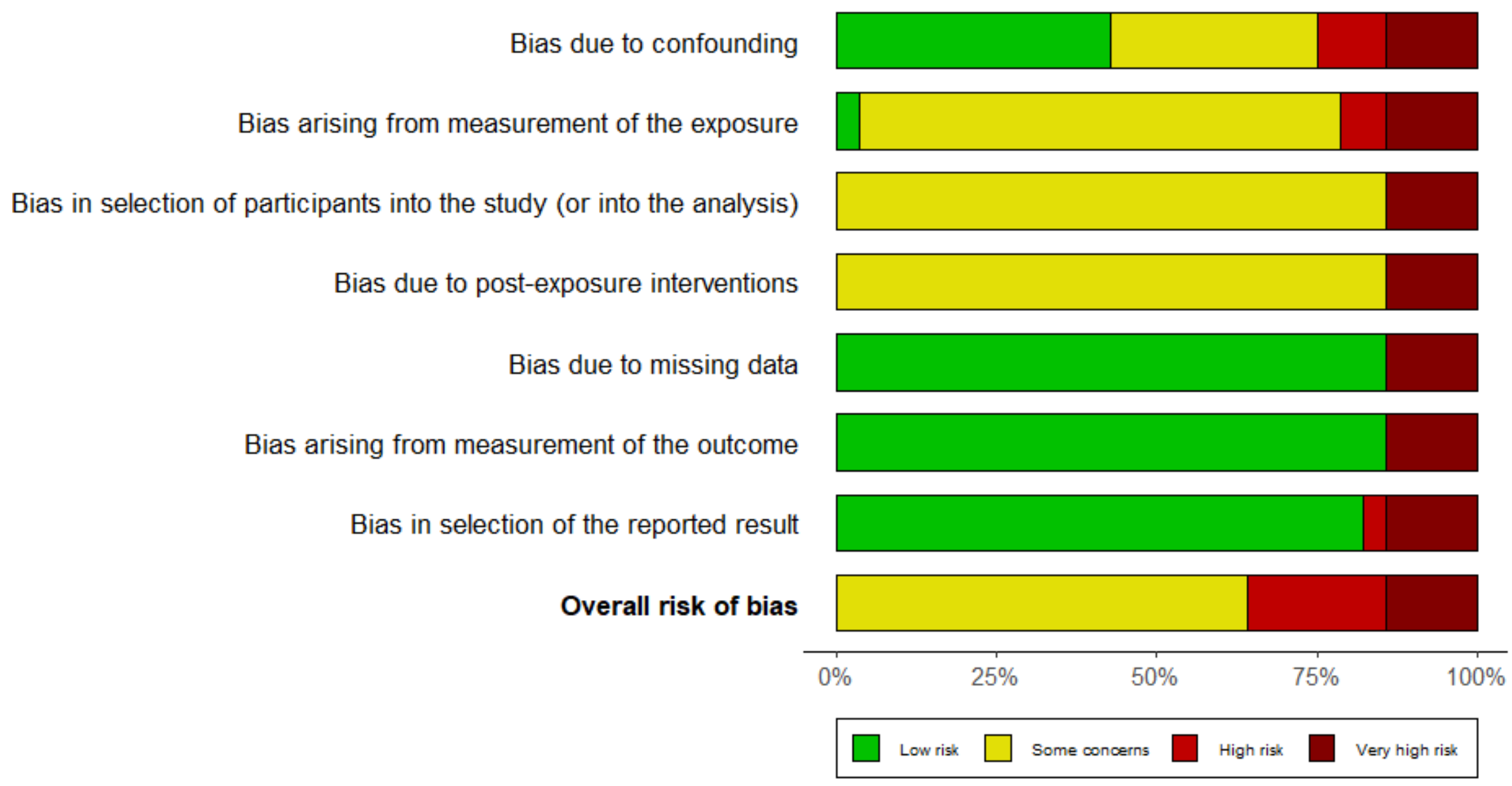

eFigure 1. Overall Risk of Bias of Included Studies Assesed Using the Risk of Bias in Non-randomized Studies of Exposures Tool

© 2026 Kefale B et al. *JAMA Network Open*.

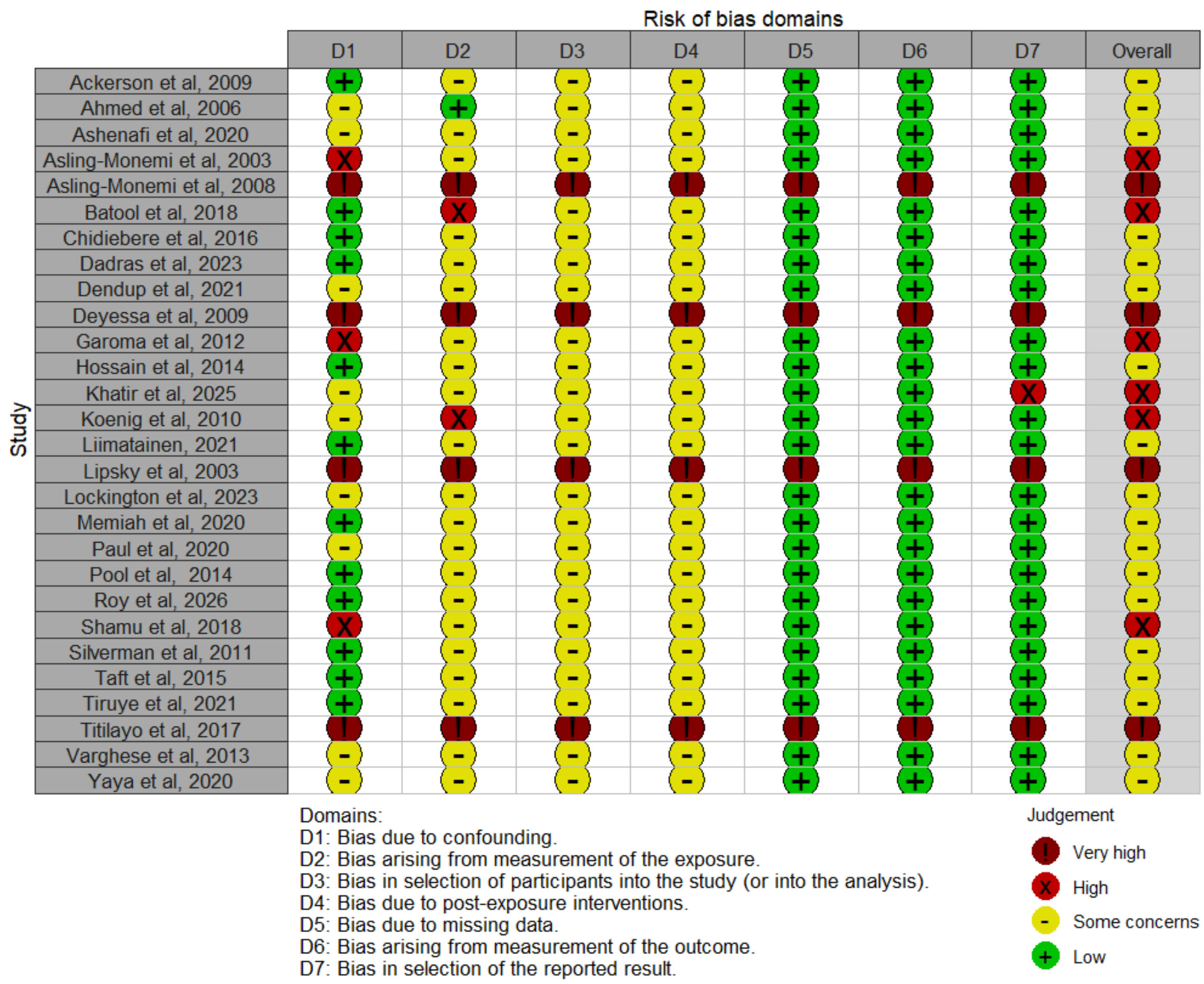

eFigure 2. Risk of Bias of Each Included Studies Assesed Using the Risk of Bias in Non-randomized Studies of Exposures Tool

© 2026 Kefale B et al. *JAMA Network Open*.

A.

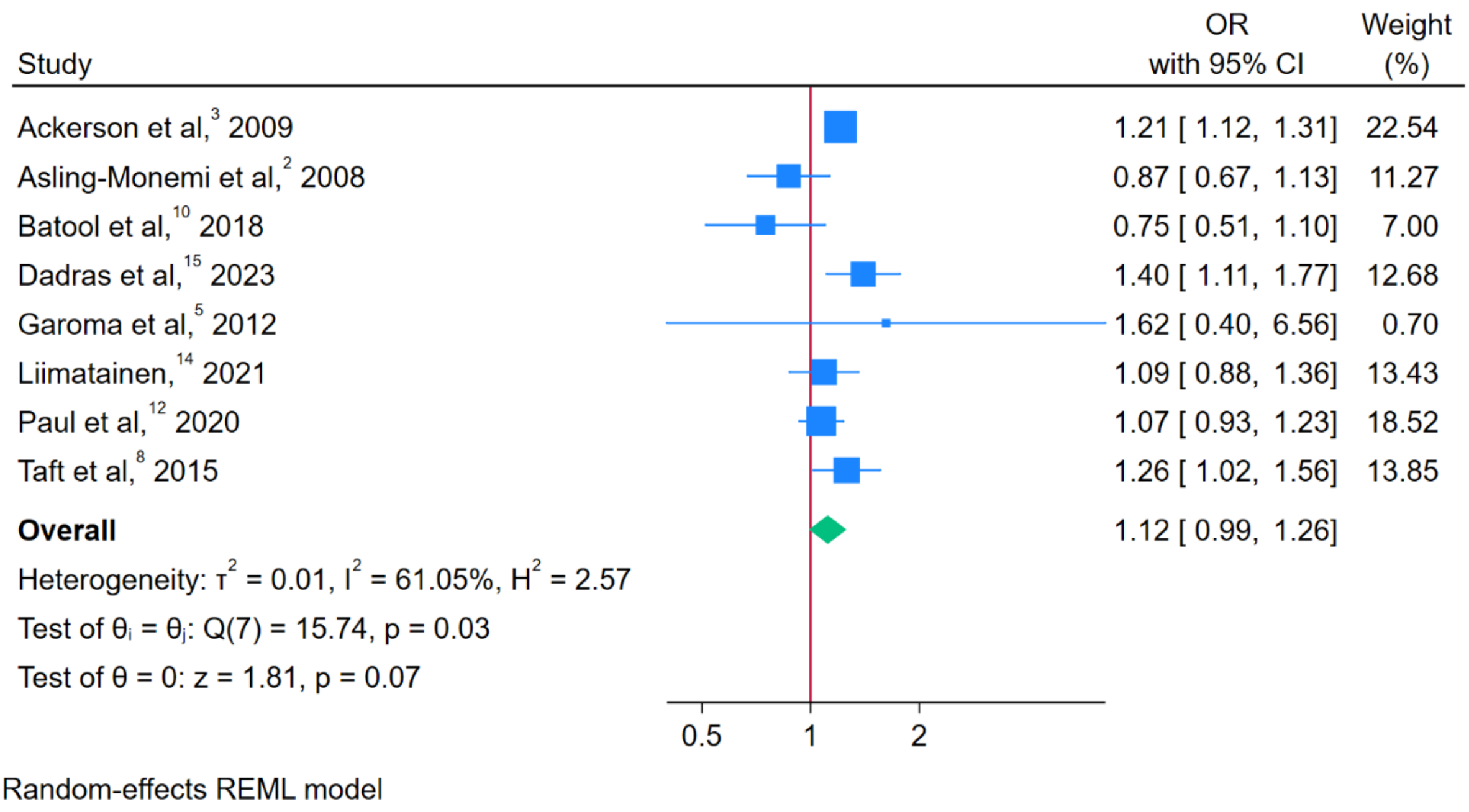

B.

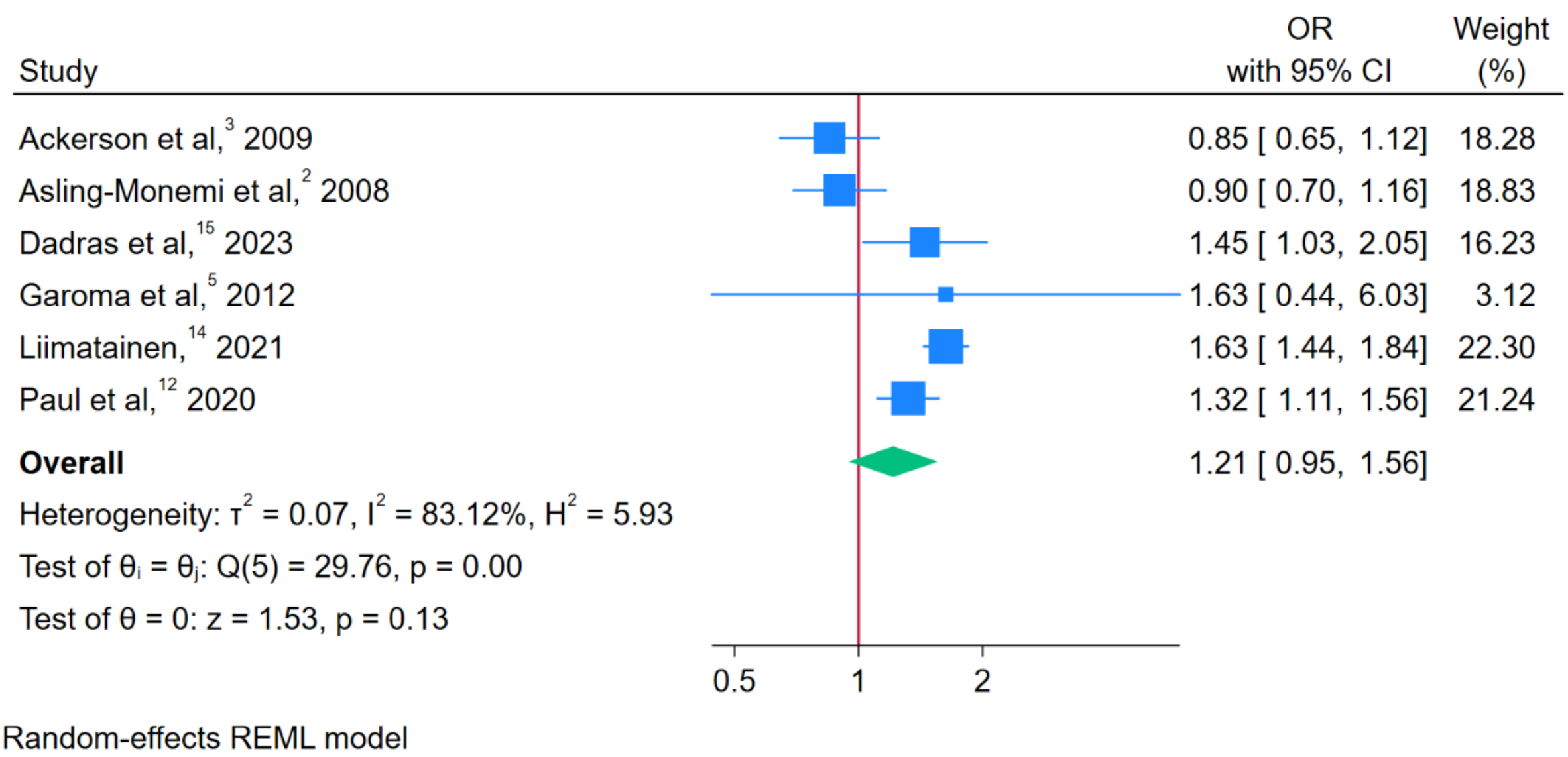

© 2026 Kefale B et al. *JAMA Network Open*.

C.

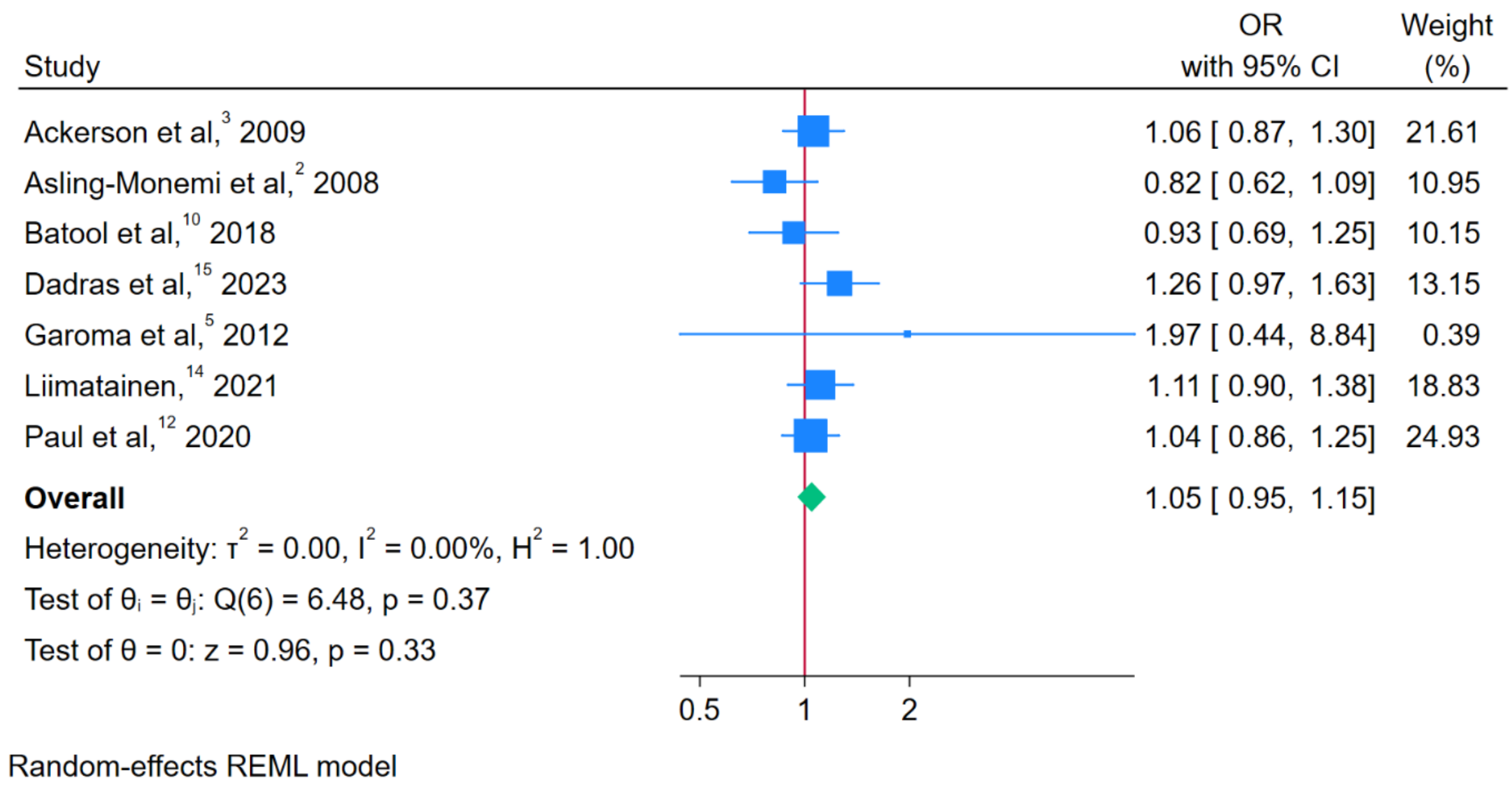

eFigure 3. Forest Plots of the Associations Between Physical (A), Sexual (B), and Emotional (C) Intimate Partner Violence with Under-Five Mortality

CI, confidence interval; OR, odds ratio; REML, restricted maximum likelihood.

© 2026 Kefale B et al. *JAMA Network Open*.

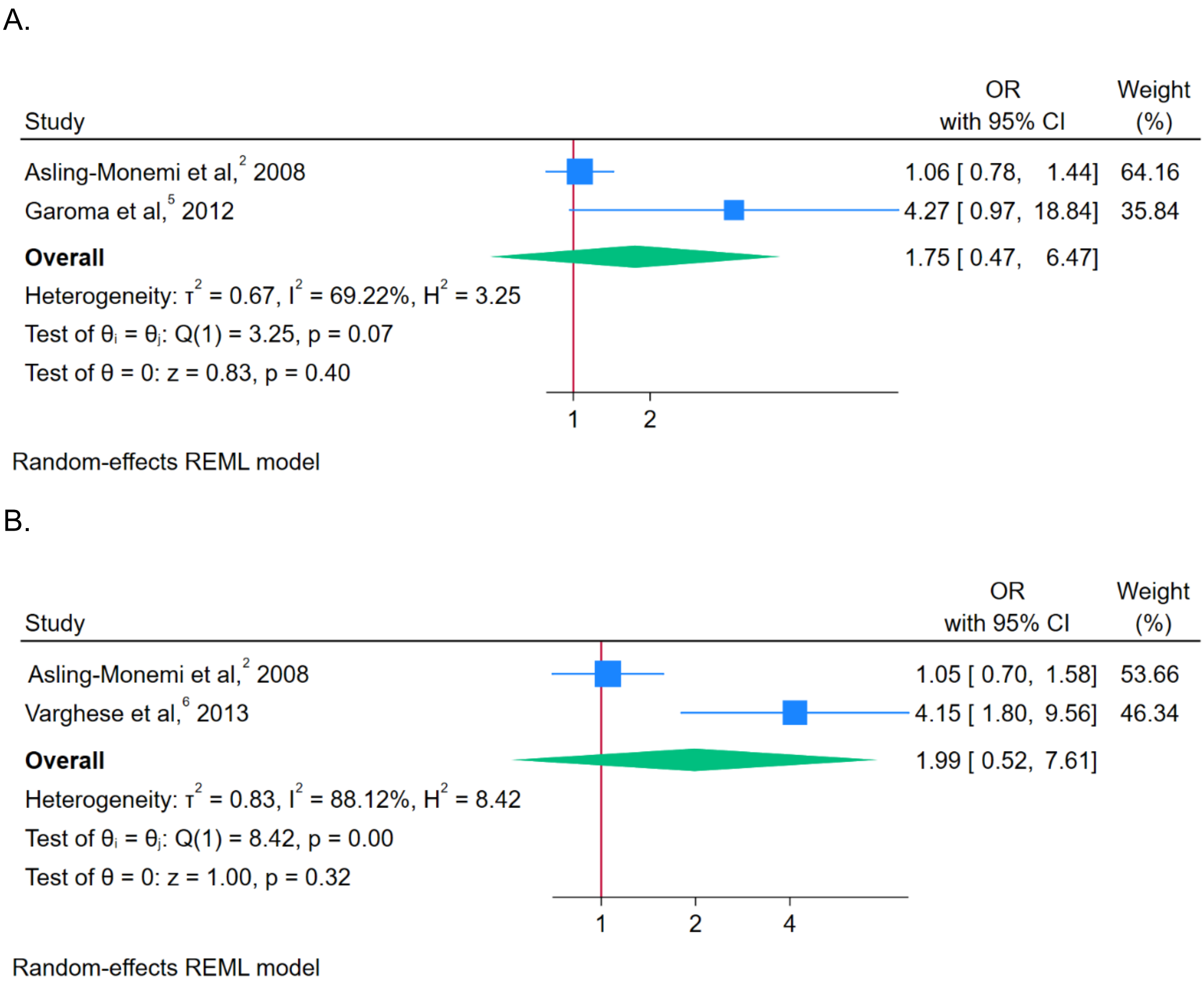

eFigure 4. Forest Plots of the Associations Between Partner Control Behavior (A) and Intimate Partner Violence During Pregnancy (B) and Under-Five Mortality

CI, confidence interval; OR, odds ratio; REML, restricted maximum likelihood.

© 2026 Kefale B et al. *JAMA Network Open*.

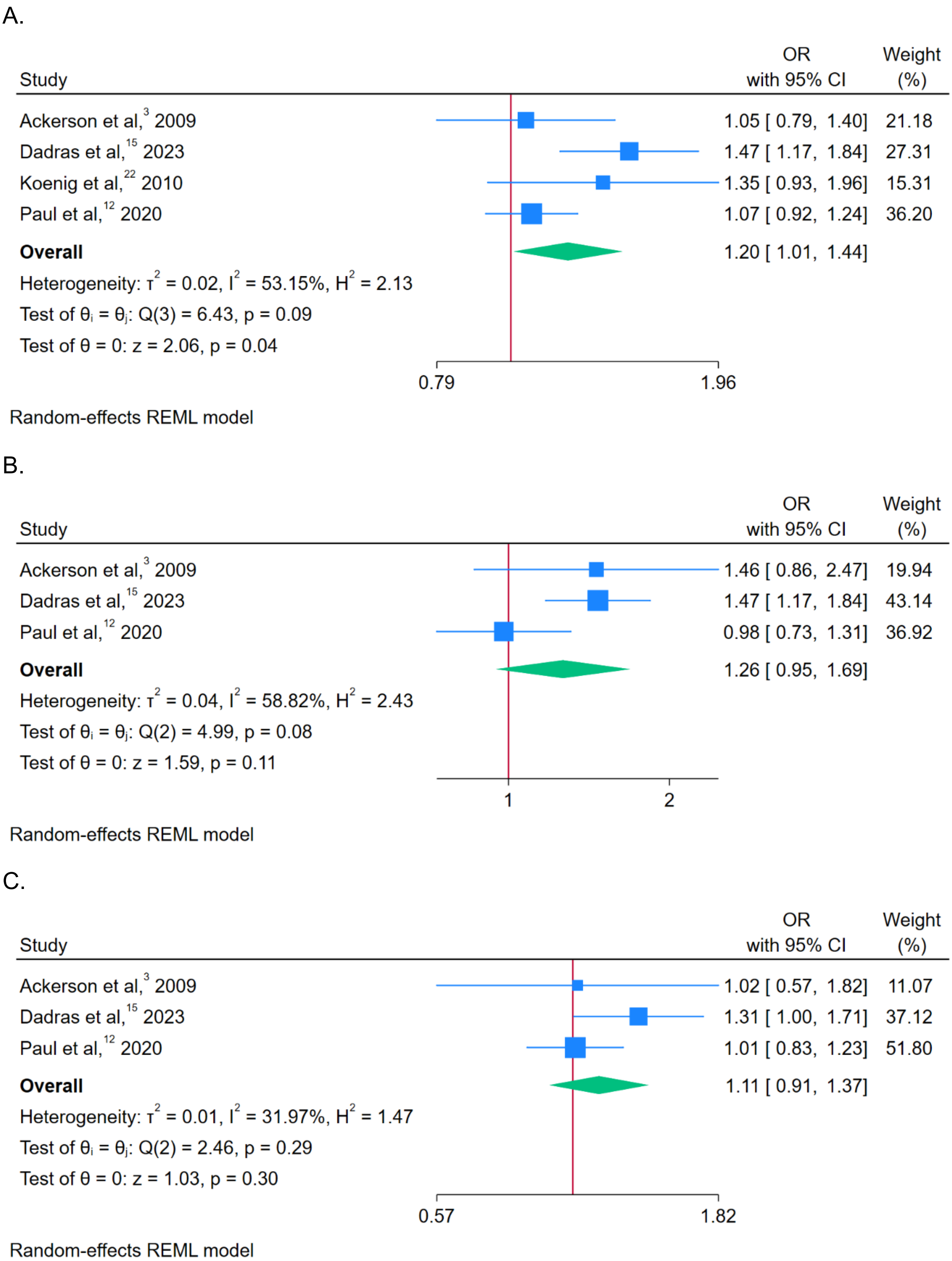

eFigure 5. Forest Plots of the Associations Between Physical (A), Sexual (B), and Emotional (C) Intimate Partner Violence with Infant Mortality

CI, confidence interval; OR, odds ratio; REML, restricted maximum likelihood.

© 2026 Kefale B et al. *JAMA Network Open*.

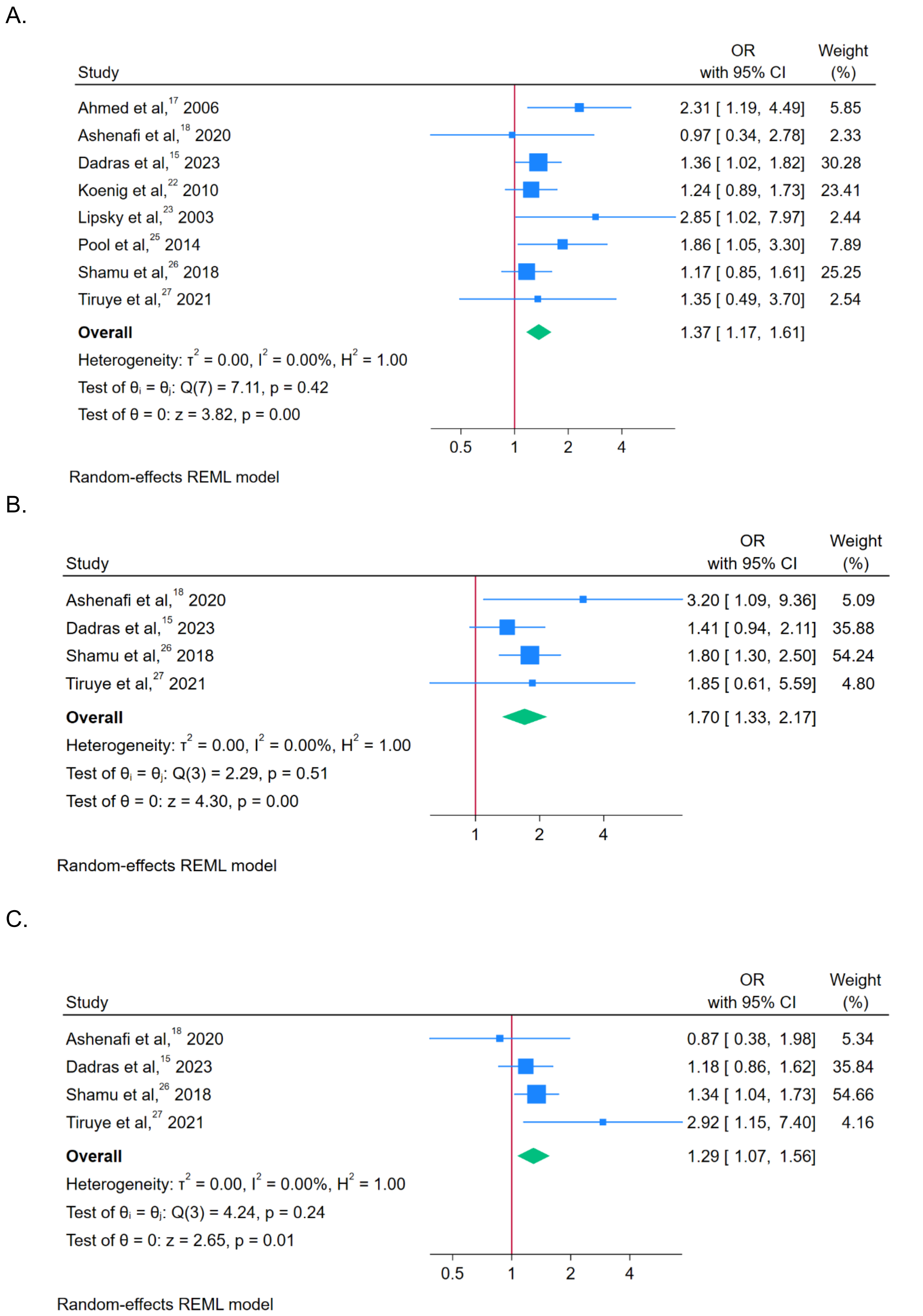

eFigure 6. Forest Plots of the Associations Between Physical (A), Sexual (B), and Emotional (C) Intimate Partner Violence with Neonatal Mortality

CI, confidence interval; OR, odds ratio; REML, restricted maximum likelihood.

© 2026 Kefale B et al. *JAMA Network Open*.

A.

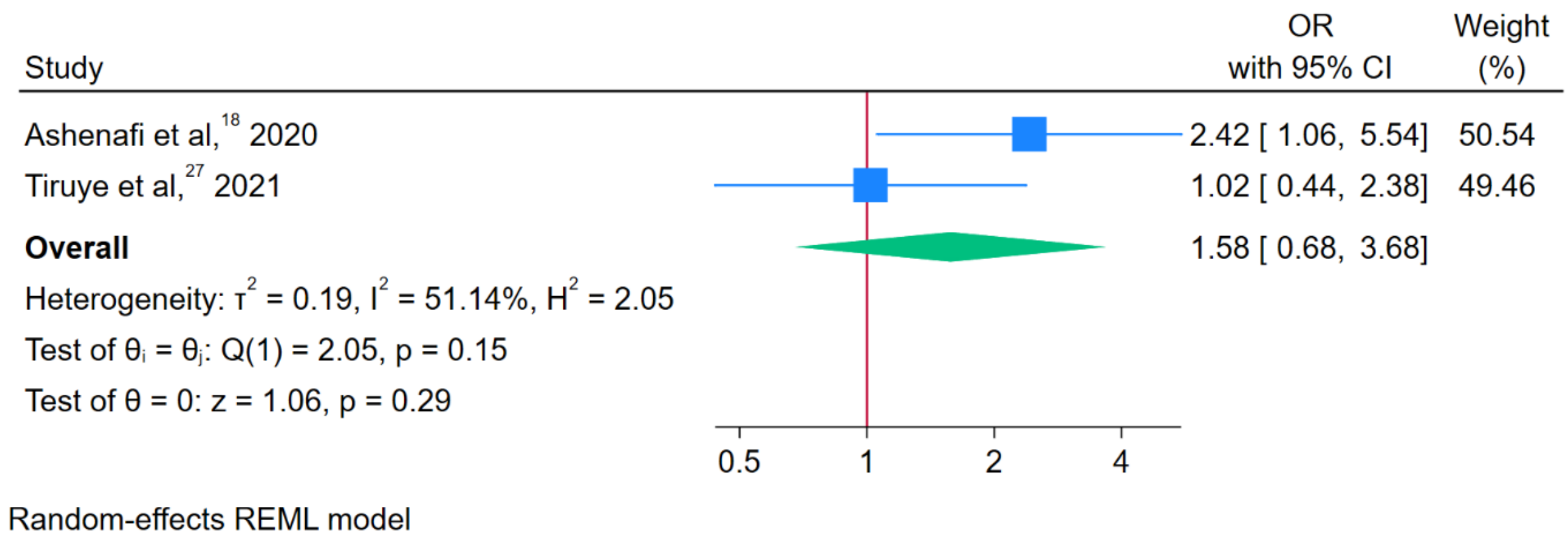

B.

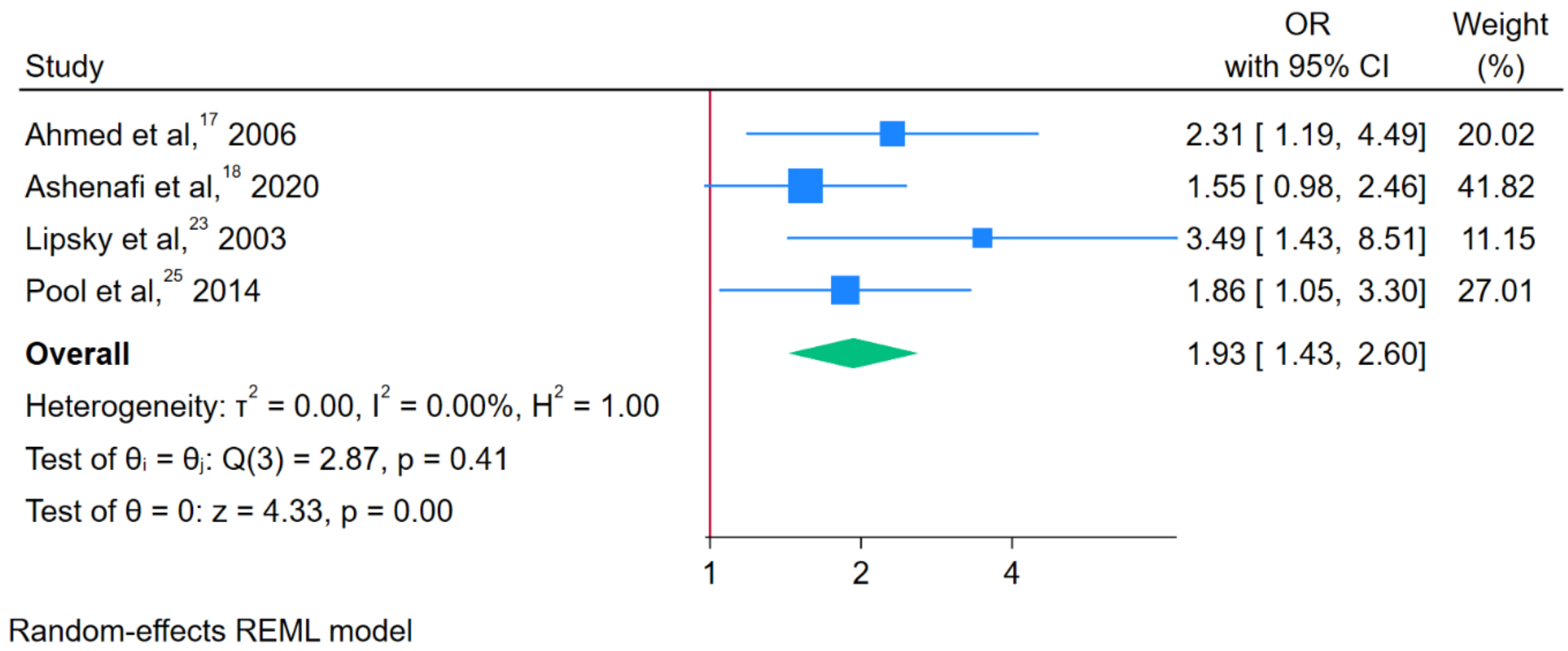

eFigure 7. Forest Plots of the Associations Between Partner Control Behavior (A) and Intimate Partner Violence During Pregnancy (B) and Neonatal Mortality

CI, confidence interval; OR, odds ratio; REML, restricted maximum likelihood.

© 2026 Kefale B et al. *JAMA Network Open*.

A.

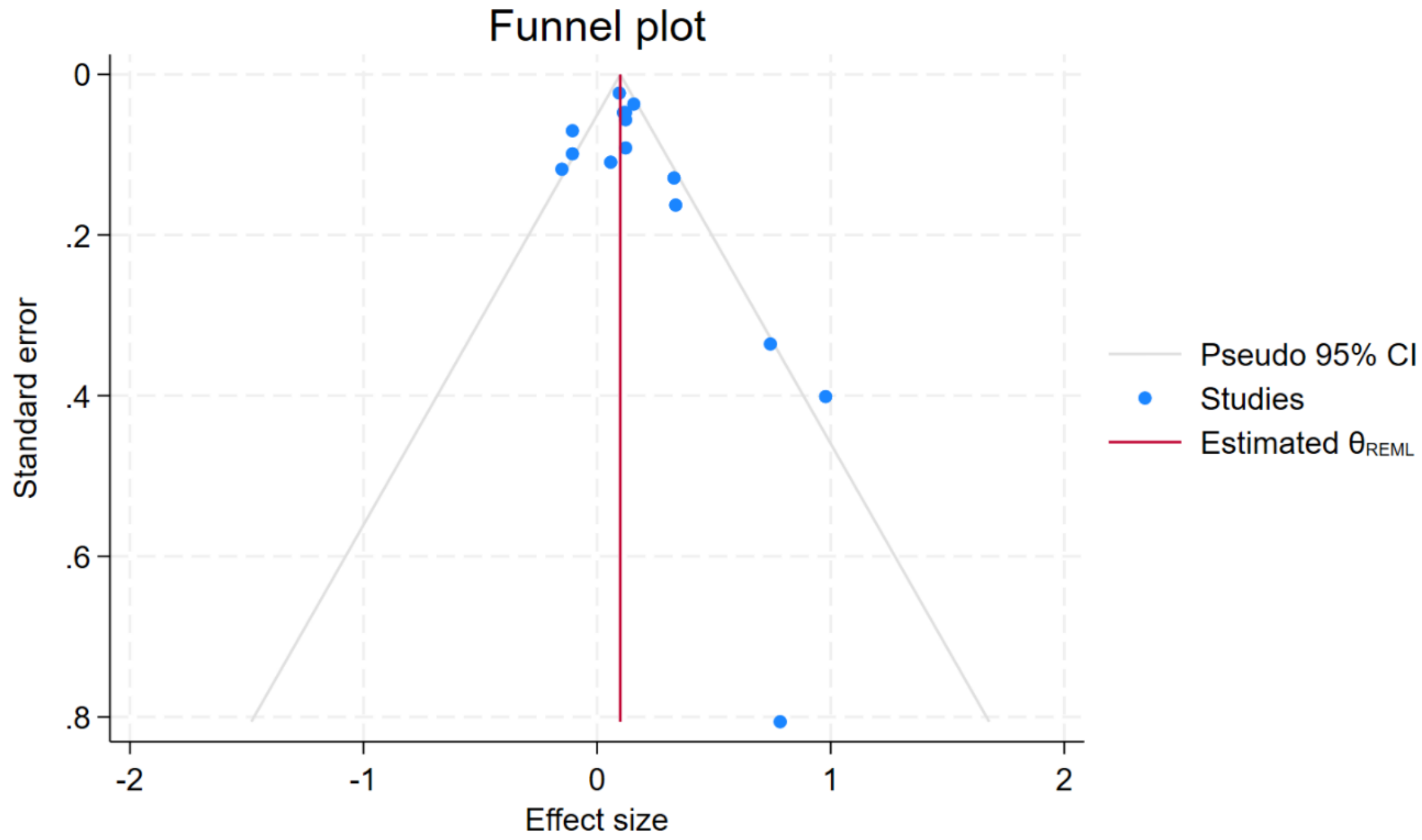

B.

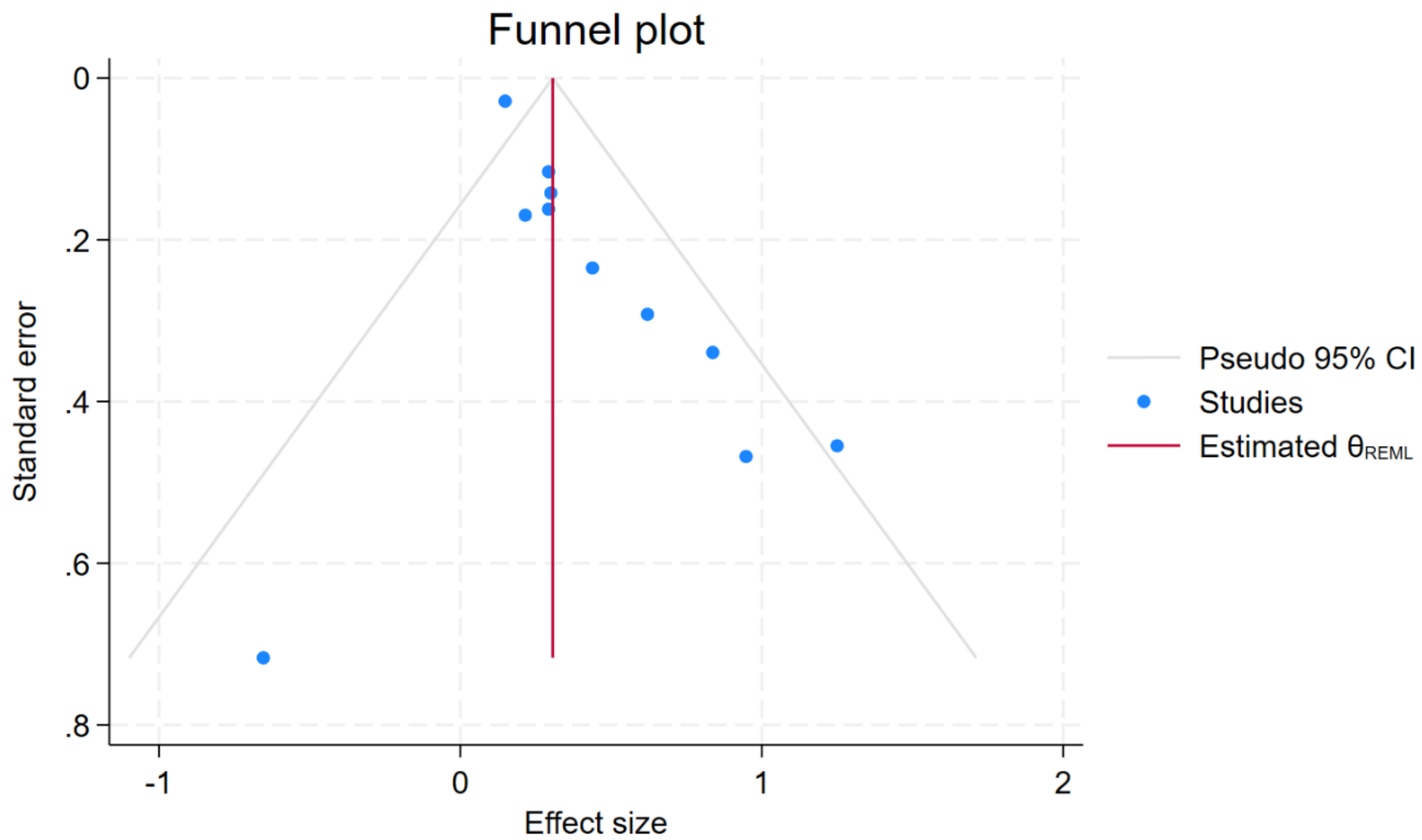

eFigure 8. Funnel Plots in Assessing Publication Bias in Studies of Intimate Partner Violence and Under-five Mortality (A) and Neonatal Mortality (B)

CI, confidence interval; REML, restricted maximum likelihood.

© 2026 Kefale B et al. *JAMA Network Open*.

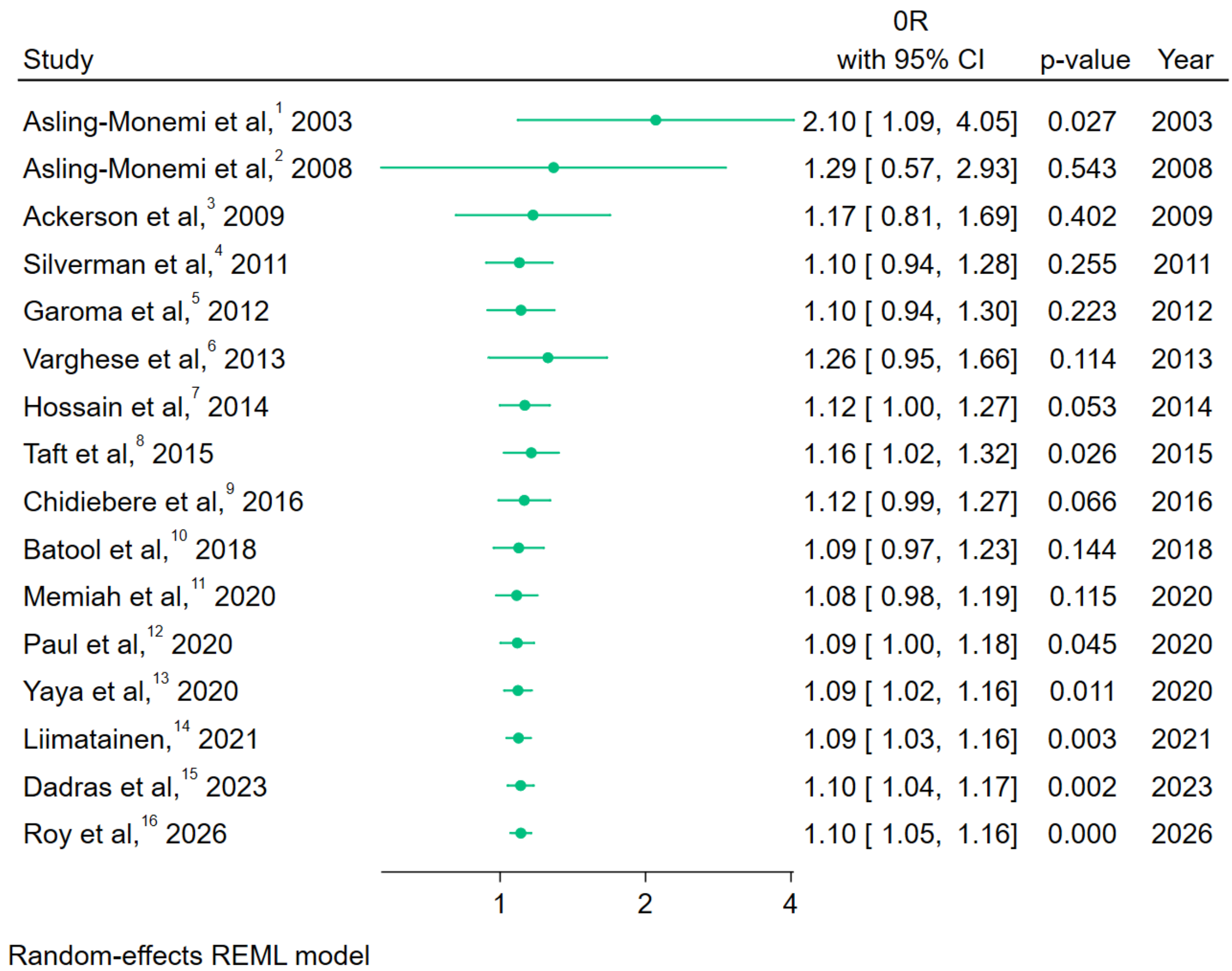

eFigure 9. Cumulative Meta-Analysis of the Association Between Any Intimate Partner Violence and Under-Five Mortality

CI, confidence interval; OR, odds ratio; REML, restricted maximum likelihood.

© 2026 Kefale B et al. *JAMA Network Open*.

A.

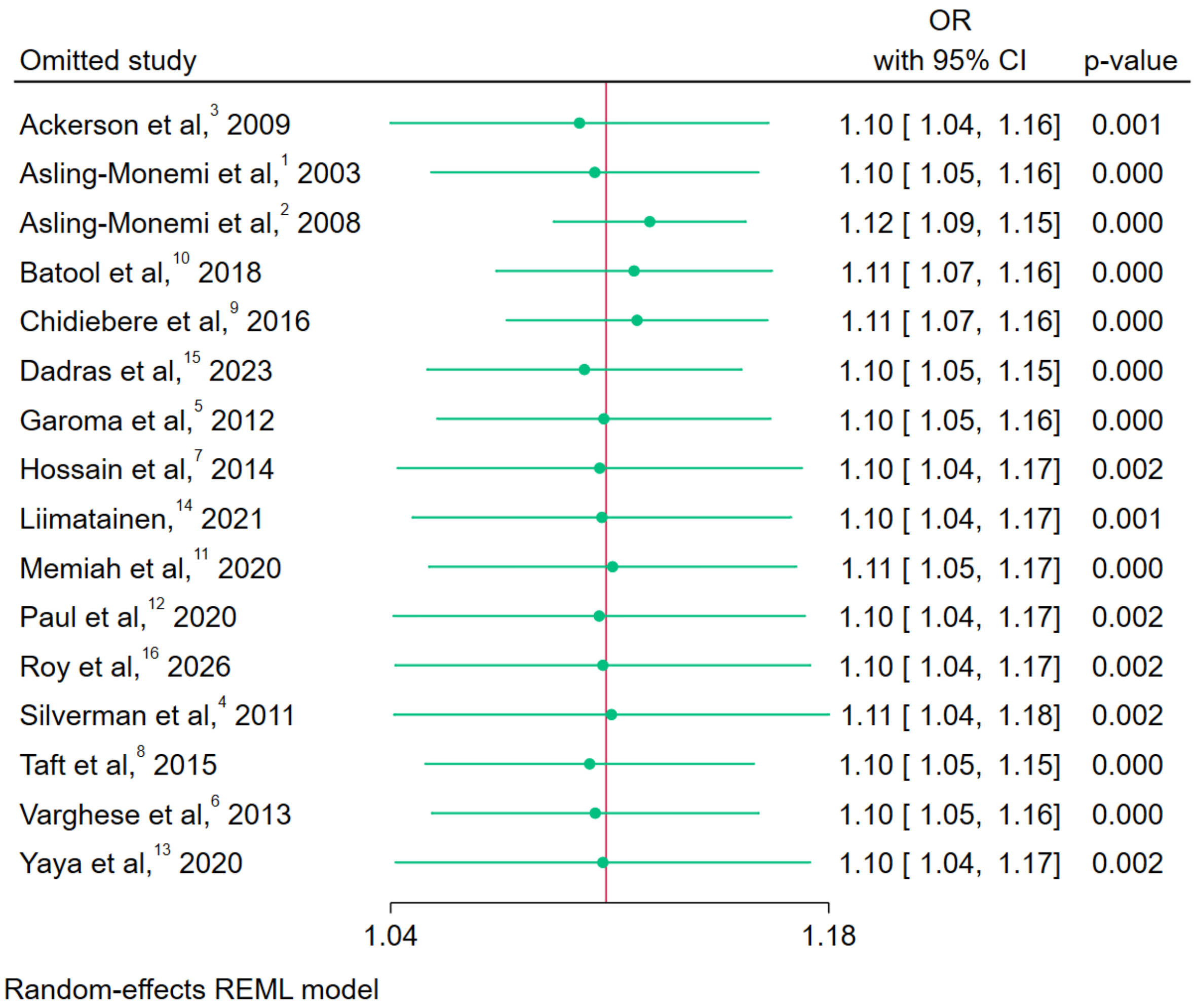

B.

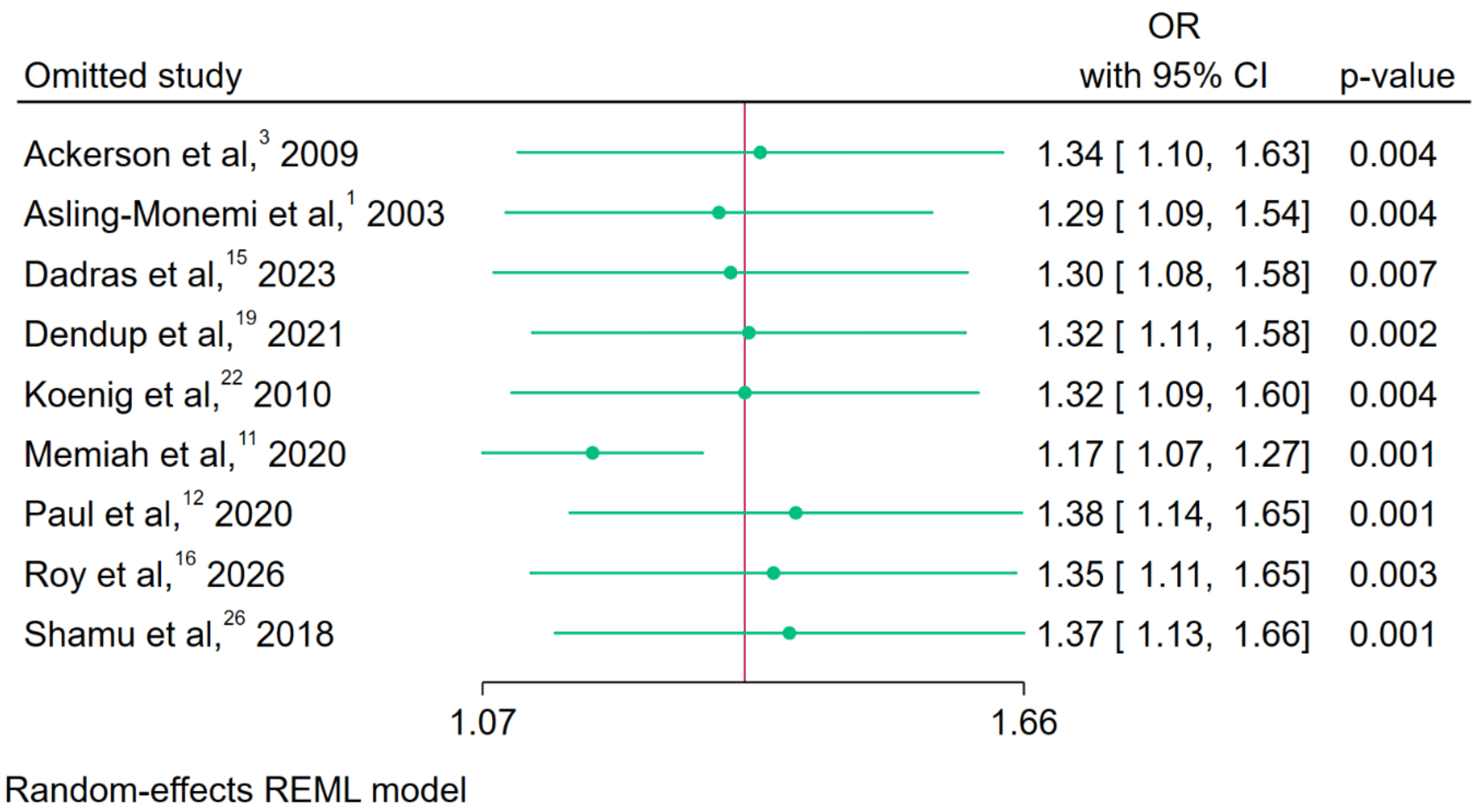

© 2026 Kefale B et al. *JAMA Network Open*.

C.

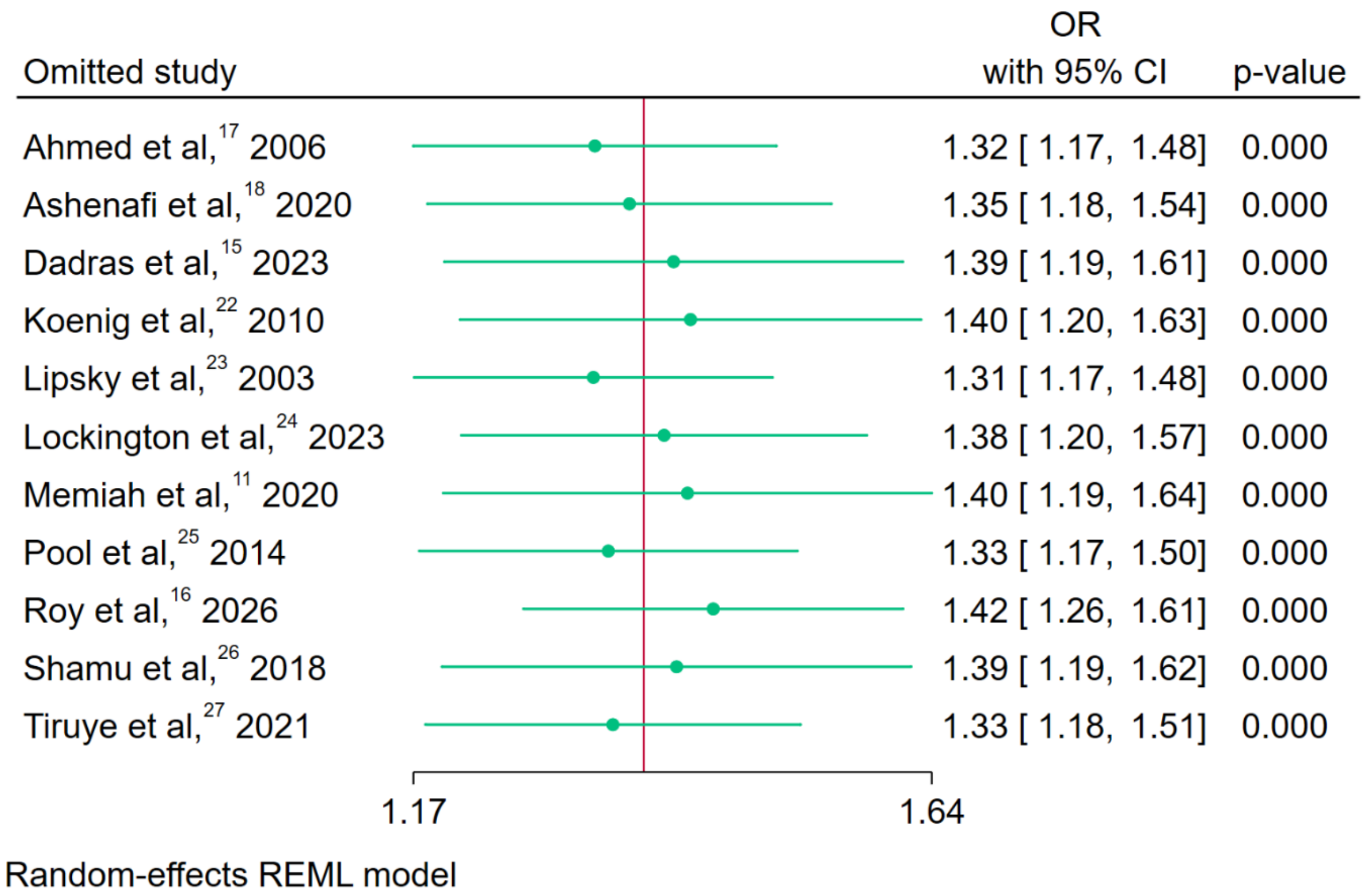

eFigure 10. Leave-One-Out Sensitivity Analysis of the Association Between Any Intimate Partner Violence And Under-Five (A), Infant (B), and Neonatal Mortality (C)

CI, confidence interval; OR, odds ratio; REML, restricted maximum likelihood.

© 2026 Kefale B et al. *JAMA Network Open*.

eReferences

1. Asling-Monemi K, Peña R, Ellsberg MC, Persson LA. Violence against women increases the risk of infant and child mortality: a case-referent study in Nicaragua. *Bull World Health Organ*. 2003;81(1):10-16.
2. Asling-Monemi K, Tabassum Naved R, Persson LA. Violence against women and the risk of under-five mortality: analysis of community-based data from rural Bangladesh. *Acta Paediatr*. 2008;97(2):226-232. doi:10.1111/j.1651-2227.2007.00597.x
3. Ackerson LK, Subramanian SV. Intimate partner violence and death among infants and children in India. *Pediatrics*. 2009;124(5):e878-889. doi:10.1542/peds.2009-0524
4. Silverman JG, Decker MR, Cheng DM, et al. Gender-based disparities in infant and child mortality based on maternal exposure to spousal violence: the heavy burden borne by Indian girls. *Arch Pediatr Adolesc Med*. 2011;165(1):22-27. doi:10.1001/archpediatrics.2010.261
5. Garoma S, Fantahun M, Worku A. Maternal intimate partner violence victimization and under-five children mortality in Western Ethiopia: a case-control study. *J Trop Pediatr*. 2012;58(6):467-474. doi:10.1093/tropej/fms018
6. Varghese S, Prasad JH, Jacob KS. Domestic violence as a risk factor for infant and child mortality: a community-based case-control study from southern India. *Natl Med J India*. 2013;26(3):142-146.
7. Hossain MA, Sumi NS, Haque ME, Bari W. Consequences of intimate partner violence against women on under-five child mortality in Bangladesh. *J Interpers Violence*. 2014;29(8):1402-1417. doi:10.1177/0886260513507140
8. Taft AJ, Powell RL, Watson LF. The impact of violence against women on reproductive health and child mortality in Timor-Leste. *Aust N Z J Public Health*. 2015;39(2):177-181. doi:10.1111/1753-6405.12339
9. Chidiebere O, Amrita N, Fombong F, Ikenna N, Uchenna E, Diddy A. Domestic Violence against Women and Its Consequence on Child Mortality: Analysis from Five African Countries. *Int J Trop Dis Health*. 2016;14(3):1-11. doi:10.9734/IJTDH/2016/23855
10. Batool S, Gul I, Naz S, Sehar Un N, Husain G. Impact of spousal violence on child health and life prospects: Evidence from Pakistan demographic health survey 2012-13. *Pak Paed J*. 2018;42(3):202-207.
11. Memiah P, Bond T, Opanga Y, et al. Neonatal, infant, and child mortality among women exposed to intimate partner violence in East Africa: a multi-country analysis. *BMC Womens Health*. 2020;20(1):10. doi:10.1186/s12905-019-0867-2
12. Paul P, Mondal D. Maternal experience of intimate partner violence and its association with morbidity and mortality of children: Evidence from India. *PLoS One*. 2020;15(4):e0232454. doi:10.1371/journal.pone.0232454
13. Yaya S, Anjorin SS, Adenini SA. Intimate partner violence, contextual factors and under-5 mortality: a multilevel analysis of cross-sectional surveys from 20 Sub-Saharan African countries. BMJ Glob Health. 2020;5(12). doi:10.1136/bmjgh-2020-003531.
14. Liimatainen M. The association between intimate partner violence and under 5-child mortality in Nigeria: A cross-sectional study based on Nigerian demographic health survey from 2018. Unpublished thesis. Uppsala, Sweden: Uppsala University; 2021. Accessed January 12, 2024. https://urn.kb.se/resolve?urn=urn:nbn:se:uu:diva-4472732021
15. Dadras O, Hazratzai M, Dadras F. Intimate Partner Violence and Children Under 5 Morbidities and Mortality in Afghanistan: Findings From a National Survey. *J Interpers Violence*. 2023;38(17-18):10105-10126. doi:10.1177/08862605231169774
16. Roy A, Pereira G, Dunne J, Nyadanu SD, Tessema GA. Maternal intimate partner violence and under five mortality in India: The effect modifying role of caste. *Public Health*. 2026;251:106099. doi:10.1016/j.puhe.2025.106099
17. Ahmed S, Koenig MA, Stephenson R. Effects of domestic violence on perinatal and early-childhood mortality: evidence from north India. *Am J Public Health*. 2006;96(8):1423-1428. doi:10.2105/ajph.2005.066316
18. Ashenafi W, Mengistie B, Egata G, Berhane Y. Intimate partner violence during pregnancy and neonatal mortality in Eastern Ethiopia: a population-based matched case-control study. Research Square [Preprint]. 2020. doi:10.21203/rs.3.rs-58851/v2
19. Dendup T, Putra IGNE, Tobgay T, Wangdi S, Wangchuk U. Determinants of infant mortality in Bhutan: evidence from the 2012 National Health Survey data. *J Popul Soc Stud*. 2021;29:255-273. doi:10.25133/JPSSv292021.016
20. Deyessa N, Berhane Y, Emmelin M, Ellsberg MC, Kullgren G, Högberg U. Joint effect of maternal depression and intimate partner violence on increased risk of child death in rural Ethiopia. *Arch Dis Child*. 2010;95(10):771-775. doi:10.1136/adc.2009.165571

© 2026 Kefale B et al. *JAMA Network Open*.

21. Khatir AG, Rahman N, Ariyo T, Jiang Q. Association between severity of physical intimate partner violence and neonatal mortality in Afghanistan: Survival analysis using Cox proportional hazard model. *Soc Sci Med*. 2025;378:118160. doi:10.1016/j.socscimed.2025.118160
22. Koenig MA, Stephenson R, Acharya R, Barrick L, Ahmed S, Hindin M. Domestic violence and early childhood mortality in rural India: evidence from prospective data. *Int J Epidemiol*. 2010;39(3):825-833. doi:10.1093/ije/dyq066
23. Lipsky S, Holt VL, Easterling TR, Critchlow CW. Impact of police-reported intimate partner violence during pregnancy on birth outcomes. *Obstet Gynecol*. 2003;102(3):557-564. doi:10.1016/s0029-7844(03)00573-8
24. Lockington EP, Sherrell HC, Crawford K, Rae K, Kumar S. Intimate partner violence is a significant risk factor for adverse pregnancy outcomes. *AJOG Glob Rep*. 2023;3(4):100283. doi:10.1016/j.xagr.2023.100283
25. Pool MS, Otupiri E, Owusu-Dabo E, de Jonge A, Agyemang C. Physical violence during pregnancy and pregnancy outcomes in Ghana. *BMC Pregnancy Childbirth*. 2014;14:71. doi:10.1186/1471-2393-14-71
26. Shamu S, Munjanja S, Zarowsky C, Shamu P, Temmerman M, Abrahams N. Intimate partner violence, forced first sex and adverse pregnancy outcomes in a sample of Zimbabwean women accessing maternal and child health care. *BMC Public Health*. 2018;18(1):595. doi:10.1186/s12889-018-5464-z
27. Tiruye TY, Chojenta C, Harris ML, Holliday E, Loxton D. The Role of Maternal Intimate Partner Violence Victimization on Neonatal Mortality in Ethiopia. *J Interpers Violence*. 2021;36(23-24):10938-10958. doi:10.1177/0886260519897331
28. Titilayo A, Anuodo OO, Palamuleni ME. Family type, domestic violence and under-five mortality in Nigeria. *Afr Health Sci*. 2017;17(2):538-548. doi:10.4314/ahs.v17i2.30
29. Afkhamzadeh A, Rahmani K, Yaghubi R, Ghadrdan M, Faraji O. Adverse perinatal outcomes of intimate partner violence during pregnancy. *Int J Hum Rights Healthc*. 2021;14(5):465-476. doi:10.1108/IJHRH-08-2019-0067
30. Alhusen JL, Ray E, Sharps P, Bullock L. Intimate partner violence during pregnancy: maternal and neonatal outcomes. *J Womens Health (Larchmt)*. 2015;24(1):100-106. doi:10.1089/jwh.2014.4872
31. Alio AP, Nana PN, Salihu HM. Spousal violence and potentially preventable single and recurrent spontaneous fetal loss in an African setting: cross-sectional study. *Lancet*. 2009;373(9660):318-324. doi:10.1016/s0140-6736(09)60096-9
32. Ara F, Sultana MM, Naoshin S, Sultana I, Hoq MN, Hossain ME. Sociodemographic determinants of child mortality based on mothers' attitudes toward partner violence: Evidence from Bangladesh. *Heliyon*. 2023;9(3):e13848. doi:10.1016/j.heliyon.2023.e13848
33. Baird KM, Phipps H, Javid N, Stephen de Vries B. Domestic and family violence and associated maternal and perinatal outcomes: A population-based retrospective cohort study. *Birth*. 2025;52(1):89-99. doi:10.1111/birt.12863
34. Ball H. Intimate Partner Violence Against Mothers Associated With Child Death in India. *Int Perspect Sex Reprod Health*. 2010;36(1):50-51.
35. Bari I, Sandberg J. Physical violence during pregnancy and infant mortality: evidence from Pakistan Demographic and Health Survey 2012–13. In: Proceedings of the American Public Health Association Annual Meeting; 2019. Accessed January 11, 2024. https://apha.confex.com/apha/2019/meetingapp.cgi/Paper/439254
36. Berenson AB, Wiemann CM, Wilkinson GS, Jones WA, Anderson GD. Perinatal morbidity associated with violence experienced by pregnant women. *Am J Obstet Gynecol*. 1994;170(6):1760-1766; discussion 1766-1769. doi:10.1016/s0002-9378(94)70352-3
37. Bhatti SH, Mamuna HI, Aftab M, et al. Risk factors of neonatal mortality in Faisalabad, Pakistan. *Pak J Pharm Sci*. 2017;30(2(Suppl.)):663-665.
38. Butchart A, Villaveces A. Violence against women and the risk of infant and child mortality. *Bull World Health Organ*. 2003;81(1):17-18.
39. Chen PH, Rovi S, Vega ML, Barrett T, Pan KY, Johnson MS. Birth Outcomes in Relation to Intimate Partner Violence. *J Natl Med Assoc*. 2017;109(4):238-245. doi:10.1016/j.jnma.2017.06.017
40. Coker AL, Sanderson M, Dong B. Partner violence during pregnancy and risk of adverse pregnancy outcomes. *Paediatr Perinat Epidemiol*. 2004;18(4):260-269. doi:10.1111/j.1365-3016.2004.00569.x
41. Covington DL, Hage M, Hall T, Mathis M. Preterm delivery and the severity of violence during pregnancy. *J Reprod Med*. 2001;46(12):1031-1039.
42. Deutsch SA, Loiselle CE, Hossain J, De Jong A. Social Factors Associated with Sudden Unexpected Infant Death. *Acad Pediatr*. 2025;25(8):102883. doi:10.1016/j.acap.2025.102883
43. Devineni K, Kumari SS, Sodumu N. Effects of intimate partner violence on pregnancy outcome. *J South Asian Feder Obst Gynae*. 2018;10(2):142-148. doi:10.5005/jp-journals-10006-1578
44. El Kady D, Gilbert WM, Xing G, Smith LH. Maternal and neonatal outcomes of assaults during pregnancy. *Obstet Gynecol*. 2005;105(2):357-363. doi:10.1097/01.Aog.0000151109.46641.03

© 2026 Kefale B et al. *JAMA Network Open*.

45. Eno EE, Fawole AA, Aboyeji AP, Adesina KT, Adeniran AS. Domestic violence and obstetric outcome among pregnant women in Ilorin, north central Nigeria. *Int J Gynaecol Obstet*. 2014;125(2):170-171. doi:10.1016/j.ijgo.2013.11.007
46. Fay KE, Yee LM. Birth Outcomes Among Women Affected by Reproductive Coercion. *J Midwifery Womens Health*. 2020;65(5):627-633. doi:10.1111/jmwh.13107
47. Ghotbizadeh F, Ghaemi M, Panahi Z, Eshraghi N, Piri M, Akbari R. The Association Between Intimate Partner Violence During Pregnancy and Maternal and Neonatal Outcomes: A Cross-Sectional Study. *Fertil Gynecol Androl*. 2023;3(1):e137800. doi:10.5812/fga-137800
48. Global Alliance for Nursing and Midwifery. Gender-based violence and child mortality—they're related. GANM. October 10, 2018. Accessed January 12, 2024. https://ganm.nursing.jhu.edu/gender-based-violence-and-child-mortality-theyre-related/
49. Gulliver PJ, Dixon RS. Immediate and long-term outcomes of assault in pregnancy. *Aust N Z J Obstet Gynaecol*. 2014;54(3):256-262. doi:10.1111/ajo.12175
50. Heaton TB, Forste R. Domestic violence, couple interaction and children's health in Latin America. *J Fam Violence*. 2008;23(3):183-193. doi:10.1007/s10896-007-9142-7
51. Ibrahimi S, Alamdar Yazdi A, Yusuf KK, Salihu HM. Association of Domestic Physical Violence With Feto-Infant Outcomes in Afghanistan. *Asia Pac J Public Health*. 2021;33(2-3):273-279. doi:10.1177/1010539520977333
52. Janssen PA, Holt VL, Sugg NK, Emanuel I, Critchlow CM, Henderson AD. Intimate partner violence and adverse pregnancy outcomes: a population-based study. *Am J Obstet Gynecol*. 2003;188(5):1341-1347. doi:10.1067/mob.2003.274
53. Jejeebhoy SJ. Associations between wife-beating and fetal and infant death: impressions from a survey in rural India. *Stud Fam Plann*. 1998;29(3):300-308.
54. Jofre-Bonet M, Rossello-Roig M, Serra-Sastre V. Intimate partner violence and children's health outcomes. *SSM Popul Health*. 2024;25:101611. doi:10.1016/j.ssmph.2024.101611
55. Karamagi CA, Tumwine JK, Tylleskar T, Heggenhougen K. Intimate partner violence and infant morbidity: evidence of an association from a population-based study in eastern Uganda in 2003. *BMC Pediatr*. 2007;7:34. doi:10.1186/1471-2431-7-34
56. Koirala S. Intimate Partner Violence and Adverse Pregnancy Outcomes. *Kathmandu Univ Med J (KUMJ)*. 2022;20(79):290-294.
57. McClintock HF, Edmonds SE, Lambert AR. Intimate partner violence and child loss: an evaluation of 7 sub-Saharan African countries. *Afr Health Sci*. 2023;23(1):276-285. doi:10.4314/ahs.v23i1.30
58. McDougal L, Strathdee SA, Rangel G, et al. Adverse pregnancy outcomes and sexual violence among female sex workers who inject drugs on the United States-Mexico border. *Violence Vict*. 2013;28(3):496-512. doi:10.1891/0886-6708.11-00129
59. Melaku G, Belay Getahun S, Abebe M, Shumye S, Semagn S. The Association Between Domestic Violence and Adverse Pregnancy Outcomes in Pregnant Women Seeking Antenatal Care at Public Hospitals in Gedeo Zone: A Prospective Cohort Study. *J Multidiscip Healthc*. 2023;16:1833-1843. doi:10.2147/jmdh.S419473
60. Menon S. Unfinished lives: the effect of domestic violence on neonatal and infant mortality. ISER Working Paper Series. No. 2014-27. Colchester, UK: Institute for Social and Economic Research (ISER), University of Essex; 2014. Accessed January 10, 2024. https://www.econstor.eu/bitstream/10419/126472/1/796389756.pdf
61. Musa A, Chojenta C, Loxton D. The association between intimate partner violence and low birth weight and preterm delivery in eastern Ethiopia: Findings from a facility-based study. *Midwifery*. 2021;92:102869. doi:10.1016/j.midw.2020.102869
62. Nassaka MG. Influence of socioeconomic status components on gender-based violence and infant mortality in Uganda. Unpublished thesis. Kampala, Uganda: Makerere University; 2023. Accessed January 10, 2024. http://hdl.handle.net/20.500.12281/16721
63. Nwachuku DN. Infant and child mortality and related religio-cultural violence against women. *J Constr Theol*. 2001;7(2):85-107.
64. Okenwa L, Lawoko S, Jansson B. Contraception, reproductive health and pregnancy outcomes among women exposed to intimate partner violence in Nigeria. *Eur J Contracept Reprod Health Care*. 2011;16(1):18-25. doi:10.3109/13625187.2010.534515
65. Okunola TO, Awoleke JO, Olofinbiyi BA, Rosiji BO, Omoya S, Olubiyi AO. Adverse birth outcomes among women exposed to intimate partner violence in pregnancy in Ikere-Ekiti, South-west Nigeria: A prospective cohort study. *Eur J Obstet Gynecol Reprod Biol*. 2021;267:186-191. doi:10.1016/j.ejogrb.2021.11.017
66. Osuorah D, Antai D, Ezeudu C, Chukwujekwu E. Effect of maternal exposure to intimate partner violence on under-five mortality in Nigeria. *Niger J Paed*. 2012;39(3):97-104. doi:10.4314/njp.v39i3.2

© 2026 Kefale B et al. *JAMA Network Open*.

67. Pandey S, Lin Y. Adjusted effects of domestic violence, tobacco use, and indoor air pollution from use of solid fuel on child mortality. *Matern Child Health J*. 2013;17(8):1499-1507. doi:10.1007/s10995-012-1163-z
68. Pastor-Moreno G, Ruiz-Pérez I, Henares-Montiel J, Petrova D. Intimate partner violence during pregnancy and risk of fetal and neonatal death: a meta-analysis with socioeconomic context indicators. *Am J Obstet Gynecol*. 2020;222(2):123-133.e125. doi:10.1016/j.ajog.2019.07.045
69. Rao N, Norris Turner A, Harrington B, Nampandeni P, Banda V, Norris A. Correlations between intimate partner violence and spontaneous abortion, stillbirth, and neonatal death in rural Malawi. *Int J Gynaecol Obstet*. 2017;138(1):74-78. doi:10.1002/ijgo.12173
70. Rawlings S, Siddique Z. Domestic violence and child mortality in the developing world. *Oxf Bull Econ Stat*. 2020;82(4):723-750. doi:10.1111/obes.12357
71. Rico E, Fenn B, Abramsky T, Watts C. Associations between maternal experiences of intimate partner violence and child nutrition and mortality: findings from Demographic and Health Surveys in Egypt, Honduras, Kenya, Malawi and Rwanda. *J Epidemiol Community Health*. 2011;65(4):360-367. doi:10.1136/jech.2008.081810
72. Sarkar NN. The impact of intimate partner violence on women's reproductive health and pregnancy outcome. *J Obstet Gynaecol*. 2008;28(3):266-271. doi:10.1080/01443610802042415
73. Sarkar NN. The cause and consequence of domestic violence on pregnant women in India. *J Obstet Gynaecol*. 2013;33(3):250-253. doi:10.3109/01443615.2012.747493
74. Schuiling KD. Disrespect and abuse of women during childbirth: a significant factor in the efforts to reduce maternal and perinatal morbidity and mortality. *Int J Childbirth*. 2015;6(1):2. doi:10.1891/2156-5287.6.1.2
75. Shelton JD, Adetunji J. Spousal violence and spontaneous fetal loss. *Lancet*. 2009;373(9674):1520. doi:10.1016/S0140-6736(09)60850-3
76. Silverman JG, Gupta J, Decker MR, Kapur N, Raj A. Intimate partner violence and unwanted pregnancy, miscarriage, induced abortion, and stillbirth among a national sample of Bangladeshi women. *Bjog*. 2007;114(10):1246-1252. doi:10.1111/j.1471-0528.2007.01481.x
77. Silverman JG, Decker MR, Gupta J, Kapur N, Raj A, Naved RT. Maternal experiences of intimate partner violence and child morbidity in Bangladesh: evidence from a national Bangladeshi sample. *Arch Pediatr Adolesc Med*. 2009;163(8):700-705. doi:10.1001/archpediatrics.2009.115
78. Sunitha HK. Domestic violence and the risk of infant and child mortality: a case-control study. Unpublished thesis. Vellore, India: Christian Medical College; 2008. Accessed January 11, 2024. https://www.semanticscholar.org/paper/Domestic-Violence-and-The-Risk-of-Infant-and-Child-Sunitha/778238af3fa42b3d3069f5d0237ae7b645fb894c
79. Tamirat KS, Sisay MM, Tesema GA, Tessema ZT. Determinants of adverse birth outcome in Sub-Saharan Africa: analysis of recent demographic and health surveys. *BMC Public Health*. 2021;21(1):1092. doi:10.1186/s12889-021-11113-z
80. Valadbeigi T, Gharaei H, Rajabi A, et al. The relationship between physical violence during pregnancy and stillbirth and neonatal mortality. *J Adv Pharm Educ Res*. 2017;7(4):450-459.
81. Wassie ST, Ejigu AG, Tilahun AG, Lambyo SHM. The impact of intimate partner violence on adverse birth outcomes in public health facilities. A prospective cohort study. *Midwifery*. 2023;126:103815. doi:10.1016/j.midw.2023.103815
82. Weitzman A, Smith-Greenaway E. The Marital Implications of Bereavement: Child Death and Intimate Partner Violence in West and Central Africa. *Demography*. 2020;57(1):347-371. doi:10.1007/s13524-019-00846-7
83. Yadav AK, Yadav J, Awasthi S, Gautam S. A Study on Relationship between Domestic Violence and Childhood Mortality in India: Using Weibull frailty Modelling Approach. *J Clin Diagn Res*. 2017;11(12):SC05-SC10. doi:10.7860/JCDR/2017/27670.10980
84. Yost NP, Bloom SL, McIntire DD, Leveno KJ. A prospective observational study of domestic violence during pregnancy. *Obstet Gynecol*. 2005;106(1):61-65. doi:10.1097/01.AOG.0000164468.06070.2a
85. Yu H, Jiang X, Bao W, Xu G, Yang R, Shen M. Association of intimate partner violence during pregnancy, prenatal depression, and adverse birth outcomes in Wuhan, China. *BMC Pregnancy Childbirth*. 2018;18(1):469. doi:10.1186/s12884-018-2113-6
86. Zakar R, Nasrullah M, Zakar MZ, Ali H. The association of intimate partner violence with unintended pregnancy and pregnancy loss in Pakistan. *Int J Gynaecol Obstet*. 2016;133(1):26-31. doi:10.1016/j.ijgo.2015.09.009

© 2026 Kefale B et al. *JAMA Network Open*.
