# Supplementary material for: Intimate Partner Violence Against Women and Mortality Among Children Younger Than 5 Years: A Systematic Review and Meta-Analysis
Source: JAMA Netw Open. 2026 Sep 2;9(9):e2631794. doi: 10.1001/jamanetworkopen.2026.31794 (PMC13539397; doi:10.1001/jamanetworkopen.2026.31794)
Supplement: Supplementary file 2 [file jamanetwopen-e2631794-s002.pdf]

Data Sharing Statement

Kefale. Intimate Partner Violence Against Women and Childhood Mortality. *JAMA Netw Open*. Published September 02, 2026. doi:10.1001/jamanetworkopen.2026.31794

Data
Data available: No

Additional Information
Explanation for why data not available: No participant data was collected for this manuscript. All data will be available within the manuscript and its supplementary file and has been collected by the respective authors of papers included in the systemic review and meta-analysis
